# Biodegradable, three-dimensional colorimetric fliers for environmental monitoring

**DOI:** 10.1126/sciadv.ade3201

**Published:** 2022-12-23

**Authors:** Hong-Joon Yoon, Geumbee Lee, Jin-Tae Kim, Jae-Young Yoo, Haiwen Luan, Shyuan Cheng, Soohyeon Kang, Huong Le Thien Huynh, Hyeonsu Kim, Jaehong Park, Joohee Kim, Sung Soo Kwak, Hanjun Ryu, Jihye Kim, Yeon Sik Choi, Hak-Young Ahn, Junhwan Choi, Seyong Oh, Yei Hwan Jung, Minsu Park, Wubin Bai, Yonggang Huang, Leonardo P. Chamorro, Yoonseok Park, John A. Rogers

**Affiliations:** ^1^ Department of Electronic Engineering, Gachon University, Seongnam-si, Gyeonggi-do 13120, Republic of Korea.; ^2^ Querrey Simpson Institute for Bioelectronics, Northwestern University, Evanston, IL 60208, USA.; ^3^ Department of Mechanical Science and Engineering, University of Illinois, Urbana, IL 61801, USA.; ^4^ Department of Biomedical Engineering, Northwestern University, Evanston, IL 60208, USA.; ^5^ Department of Chemical and Biomolecular Engineering, University of Illinois, Urbana, IL 61801, USA.; ^6^ Center for Bionics of Biomedical Research Institute, Korea Institute of Science and Technology, Seoul 02792, Republic of Korea.; ^7^ Department of Advanced Materials Engineering, Chung-Ang University, 4726 Seodong-daero, Daedeok-myeon, Anseong-si, Gyeonggi-do 17546, Republic of Korea.; ^8^ Department of Materials Science and Engineering, Yonsei University, 50 Yonsei-ro, Seodaemun-gu, Seoul 03722, Republic of Korea.; ^9^ Department of Chemical Engineering, Dankook University, Yongin 16890, Republic of Korea.; ^10^ Department of Electronic Engineering, Hanyang University, Seoul, Republic of Korea.; ^11^ Department of Applied Physical Sciences, University of North Carolina at Chapel Hill, Chapel Hill, NC 27514, USA.; ^12^ Department of Mechanical Engineering, Northwestern University, Evanston, IL 60208, USA.; ^13^ Department of Civil and Environmental Engineering, Northwestern University, Evanston, IL 60208, USA.; ^14^ Department of Materials Science and Engineering, Northwestern University, Evanston, IL 60208, USA.; ^15^ Department of Advanced Materials Engineering for Information and Electronics, Kyung Hee University, Yongin 17104, Republic of Korea.; ^16^ Department of Neurological Surgery, Northwestern University, Evanston, IL 60208, USA.; ^17^ Feinberg School of Medicine, Northwestern University, Evanston, IL 60208, USA.

## Abstract

Recently reported winged microelectronic systems offer passive flight mechanisms as a dispersal strategy for purposes in environmental monitoring, population surveillance, pathogen tracking, and other applications. Initial studies indicate potential for technologies of this type, but advances in structural and responsive materials and in aerodynamically optimized geometries are necessary to improve the functionality and expand the modes of operation. Here, we introduce environmentally degradable materials as the basis of 3D fliers that allow remote, colorimetric assessments of multiple environmental parameters—pH, heavy metal concentrations, and ultraviolet exposure, along with humidity levels and temperature. Experimental and theoretical investigations of the aerodynamics of these systems reveal design considerations that include not only the geometries of the structures but also their mass distributions across a range of bioinspired designs. Preliminary field studies that rely on drones for deployment and for remote colorimetric analysis by machine learning interpretation of digital images illustrate scenarios for practical use.

## Teaser

Three-dimensional, biodegradable, winged devices enable remote environmental monitoring.

## INTRODUCTION

High-resolution methods for fabricating three-dimensional (3D) structures create design opportunities in microsystems technologies that lie beyond those associated with conventional 2D multilayered configurations (*1*–*4*). A recent study introduced the notion of wind-dispersed seeds as the inspiration for 3D winged sensors capable of remotely monitoring environmental parameters (*1*). The work focused on structures that engage in helicopter-like motions similar to those of *Tristellateia* seeds (*5*, *6*), with wireless, electronic components for sensing (*1*). Related studies highlight additional options in electronic circuitry and in aerodynamic designs, specifically those inspired by dandelion seeds (*7*). In both cases, requirements for power sources and constraints in the range of wireless communication limit the potential applications. Considerations associated with the costs of the devices and the means for their subsequent recovery represent additional complicating factors.

Here, we report a collection of materials, 3D designs, and sensing mechanisms that address the drawbacks of these previous approaches. Specifically, we present a set of environmentally degradable materials in 3D layouts for fliers that support colorimetric chemical reagents as the basis for remote sensing of key environmental parameters—pH level (*8*), presence/concentration of heavy metals (*9*), and ultraviolet (UV) dose (*10*), along with humidity and temperature (*11*, *12*). The designs optimize the aerodynamic behaviors across a range of configurations, each evaluated quantitatively based not only on the geometries but also on the mass distributions. The schemes for forming these 3D structures extend mechanically induced buckling methods described previously by eliminating the need for bonding to a supporting substrate (*1*–*3*), thereby facilitating release into free-standing forms. Field tests illustrate application of large collections of such types of 3D colorimetric fliers with drones for deployment and for remote sensing via digital image capture and analysis based on machine learning. Degradation triggered by natural environmental processes eliminates the need for recovery after use (*13*, *14*). These collective features suggest a strong potential for realistic applications in remote, spatiotemporal monitoring.

## RESULTS

### Designs and fabrication procedures for biodegradable, colorimetric, 3D seed-inspired fliers

**Fig. 1.**
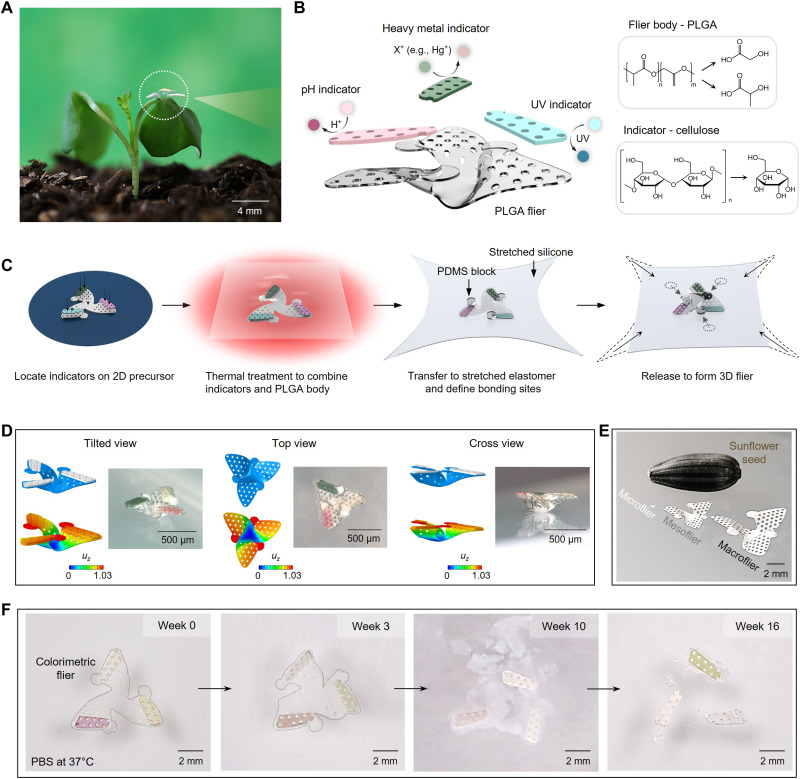
Design features of a degradable, colorimetric 3D flier for monitoring the environment. (**A**) Photograph of a representative device (diameter, about 4.2 mm; mass, 1.5 mg) resting on the leaf of a pea sprout. (**B**) Schematic illustration of the two parts of the device: (i) a body and set of wings formed in poly(lactic-co-glycolic acid) (PLGA; thickness ~60 mm) and (ii) colorimetric assays for pH, heavy metals, and UV exposure that use responsive chemical indicators supported by cellulose (thickness ~80 mm). All of the materials are environmentally benign, and they naturally biodegrade via hydrolysis and fungal activity. (**C**) Schematic illustration of the fabrication process. (**D**) Optical micrographs of a 3D colorimetric microflier at three different angled views with corresponding geometries predicted by FEA simulations. (**E**) Photograph that compares the sizes of 3D micro-, meso-, and macrofliers with a sunflower seed. (**F**) Images of dissolution of a colorimetric flier at various time points following immersion in PBS (pH 7.4) at 37°C.

The photograph in Fig. 1A shows a biodegradable, colorimetric 3D flier resting on the leaf of a plant. This radially symmetric design derives inspiration from the helicopter-type seeds of the *Tristellateia australasiae* plant, as the basis for stabilized aerial dispersal by rotational motions during free fall in air (fig. S1, design precursor) (*1*). The fabrication scheme enables mass production of these and related structures in ways that align with planar processing techniques used in the semiconductor industry (*1*). As a result, integration of wide-ranging classes of thin-film materials and micro/nanostructures (*15*–*17*), including complete integrated circuits (*1*, *3*, *7*, *18*), is readily possible. The design strategy includes considerations not only in manufacturing, aerodynamics, and function (*1*, *7*, *19*) but also in scaled distribution, as discussed subsequently (*1*, *7*).

As shown in Fig. 1B, these fliers use poly(lactide-co-glycolide) (PLGA) 75:25 (lactide:glycolide) for the body and wings (thickness ~60 μm) (*20*). Thin films of cellulose (thickness ~80 μm) that support colorimetric chemical reagents bond to the wings to allow quantitative assessment of environmental parameters via remote digital image capture and quantitative color analysis (*11*, *21*). Examples introduced here include pH (*22*), presence/concentration of heavy metals (*9*), and UV exposure dose (*23*), along with humidity and temperature (*24*, *25*). A perforated design for the cellulose and PLGA films facilitates contact bonding at temperatures slightly above the glass transition (*26*), without separate adhesive materials, in a planar geometry before transformation into the desired 3D shape (Fig. 1C; see Materials and Methods and fig. S2 for expanded view). The process involves transfer of planar PLGA/cellulose structures (i.e., 2D precursors) onto a prestretched silicone elastomer substrate (Dragon Skin, thickness ~500 μm) that supports small blocks of polydimethylsiloxane (PDMS) positioned at locations that interface with base features on the precursor. Relaxing the substrate imposes compressive stresses on the precursor to yield 3D forms through controlled buckling (*2*). This process occurs at temperatures close to the glass transition temperature of the PLGA. Cooling to room temperature and then stretching the PDMS releases the 3D structures as free-standing fliers. This scheme builds on methods reported previously (*27*) but avoidsthe need for interface adhesion between the precursors and the elastomeric substrates.

Figure 1D highlights an example of a microscale (half-widths of wings <1 mm; microfliers) colorimetric 3D flier (microflier) through optical micrographs collected at different viewing angles together with corresponding results of finite element analysis (FEA). This microflier supports three cellulose-supported colorimetric indicators, one each for monitoring pH, heavy metals, and UV. These elements create some additional curvature of the PLGA near the three sites of bonding to the PDMS substrate, consistent with predictions by FEA. The agreement between experiment and the FEA establishes the utility of computational mechanics as a robust design tool (*2*).

Figure 1E compares the sizes of 3D microfliers, mesofliers (half-widths of wings >1 and <5 mm), and macrofliers (half-widths of wings >5 mm) formed using the same approach in 3D fabrication, next to a sunflower seed as a reference. An essential characteristic of these fliers is that their constituent materials resorb harmlessly in the environment (*13*, *28*). Specifically, PLGA dissolves when exposed to water by hydrolysis into its monomers, glycolic and lactic acid (*29*). Fungi and other organisms initiate the decomposition of cellulose (*13*), which is followed by hydrolysis into glucose (*30*). Figure 1F shows photographs of a colorimetric flier collected at various times following immersion in phosphate-buffered saline (PBS; pH 7.4) solution at 37°C. The body material, PLGA, dissolves within 16 weeks. The colorimetric indicators, including the cellulose, undergo degradation over comparatively long time scales by environmental processes (details are in ”Practical applications as environmental indicators“).

### Aerodynamic characteristics

**Fig. 2.**
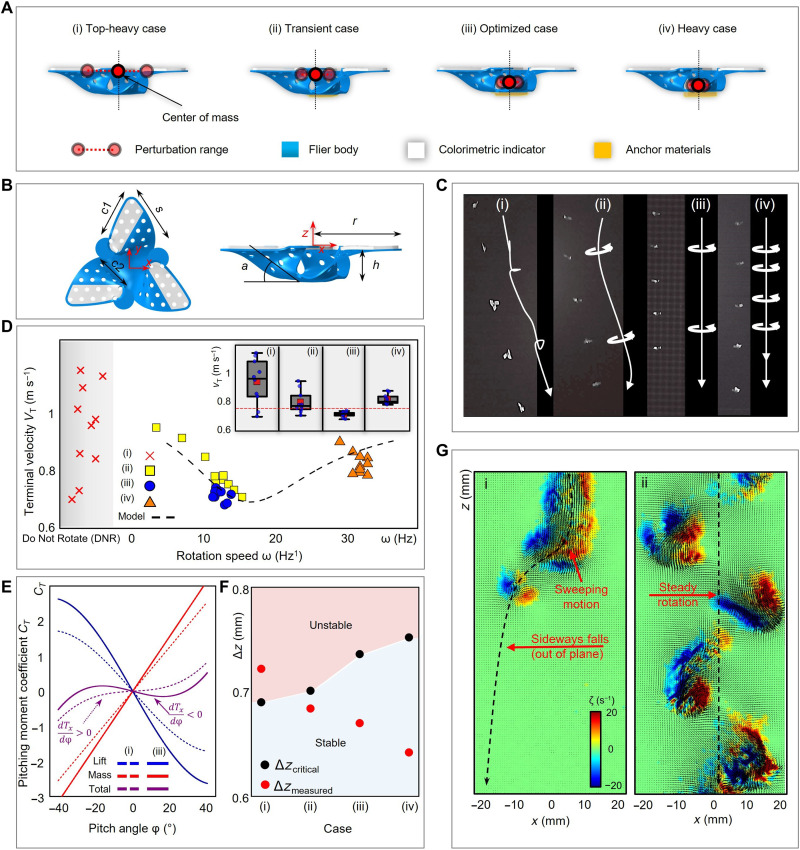
Aerodynamics of 3D colorimetric fliers. (**A**) Schematic illustrations of 3D colorimetric fliers with centers of gravity defined by different materials located at their base regions. (**B**) Schematic diagram of a 3D colorimetric flier to define keyparameters: c1, front chord; c2, real chord; *s*, wingspan; *a*, angle of attack; *r*, half-width; *h*, height. (**C**) Optical images of (i) top-heavy, (ii) middle, (iii) optimized, and (iv) heavy cases at various time points during free fall. The behaviors correspond to (i) tumbling, (ii) zigzag rotating, (iii) optimal rotating, and (iv) fast-fall rotating motions. (**D**) Measured (symbols) and computed (dashed line) terminal velocity as a function of terminal rotation speed for each of these cases [see (A)]. (**E**) Pitching moment coefficients generated from lift (blue), gravity (red), and both terms combined (purple) as a function of pitch angle. (**F**) Modeled critical, ∆*z*_critical_ (black circle), and measured (red cross), ∆*z*, center of mass height for all cases. (**G**) Representative, instantaneous flow fields associated with free fall of (i) top-heavy and (iii) optimized colorimetric fliers. Color contours denote vorticity.

Studies of the aerodynamics of these types of fliers without the colorimetric indicators appear elsewhere, in structures where the mass and geometric centers coincide. In general, the weight distributions can profoundly influence the falling behaviors. The weights and shapes of the colorimetric indicators introduced here are important to consider, as perturbations from the simple designs associated with the angle of attack and wind conditions reported previously (*1*). For a given geometry, these characteristics can change the flight dynamics from straight and stable motions to zigzag and tumbling behaviors (*31*). Colorimetric fliers with designs described above are top-heavy structures, characterized by a center of mass located high above the geometric center of the structure, ∆*z*, and a corresponding irregular tumbling flight pattern. Figure 2A illustrates how the addition of materials, which we refer to as anchor materials, below the base of the structure eliminates this undesirable behavior. FEA results that consider the geometries and masses of these anchor materials and computed positions of the centers of mass aid in the selection of optimized layouts (fig. S3).

Figure 2C exhibits four types of falling behaviors, including tumbling, zigzag rotating, optimal rotating, and fast-fall rotating motions, which result from integration of different anchor materials (see Materials and Methods). The relationship between terminal velocity, *v*_T_, and rotation speed, ω, for each case further illustrates these essential effects (Fig. 2D). The top-heavy (i) case always fails to rotate and instead exhibits a tumbling behavior. The heavy case (iv) falls 17% more quickly and rotates 2.6× faster than the optimized case (iii). Analytical modeling (Fig. 2B and fig. S4) of *v*_T_ and ω captures the underlying aerodynamics, as illustrated in Fig. 2D. The results define an anchor weight *W*_a_ that minimizes the terminal velocity for stable rotational motions. For structures with anchor weight *W* < *W*_a_, the rotation speed decreases and leads to a large effective angle of attack and a stall that prevents generation of lift. For *W* > *W*_a_, the excess weight counteracts additional lift generation, thereby increasing the terminal velocity. The stability criterion for autorotating behavior requires that the system restores to the equilibrium state after a perturbation. The static pitch stability criterion is ${\frac{dT_{x}}{d\varphi }|}_{\varphi_{0}}<0$ (notes S1 and S2 and figs. S4 to S8), where ϕ, *T_x_*, and ϕ_0_ = 0° are the pitching angle, the torque about the *x* axis, and the equilibrium pitching angle, with a sign convention that follows the right-hand rule (ϕ > 0 for counterclockwise rotation about the *x* axis). Two forces may contribute to *T_x_*, namely, the aerodynamic lift and gravitation force; the former acts through the center of each wing and the latter acts through the center of mass (fig. S5). Figure 2E shows the decomposed torque generation for case (i) (dashed lines, unstable) and case (iii) (solid lines, stable), where *T_x_* from gravity (red) is the source of instability, with a positive slope in the torque pitching angle diagram ($\frac{dT_{x,\mathrm{mass}}}{d\varphi }>0$). The critical location of the center of mass ∆*z*_critical_ (with *z* = 0 defined at the base of microflier) can be determined from the neutral stability condition for each flier design, as described with the parameter ∆*z*_measure_ in Fig. 2F. The analysis predicts the flier stability well, where the unstable case (i) corresponds to ∆*z* > ∆*z*_critical_. All other cases have ∆*z* < ∆*z*_critical_, resulting in ${\frac{dT_{x}}{d\varphi }|}_{\varphi_{0}}<0$. Complementary particle image velocimetry (PIV) experiments (detailed information appears in Materials and Methods) reveal wake dynamics of free-falling fliers with representative cases (i) and (iii) as shown in Fig. 2G. As expected, case (i) shows asymmetric, irregular wake characteristics associated with tumbling motions, while case (iii) exhibits tip vortex structures associated with autorotation. Given stable autorotation mechanisms for a given weight distribution, fluid structure interaction analyses similar to those described previously can be applied to investigate the effects of attack angles, wind conditions, and entanglements (*1*).

### Additional types of 3D fliers inspired by wind-dispersed seeds

**Fig. 3.**
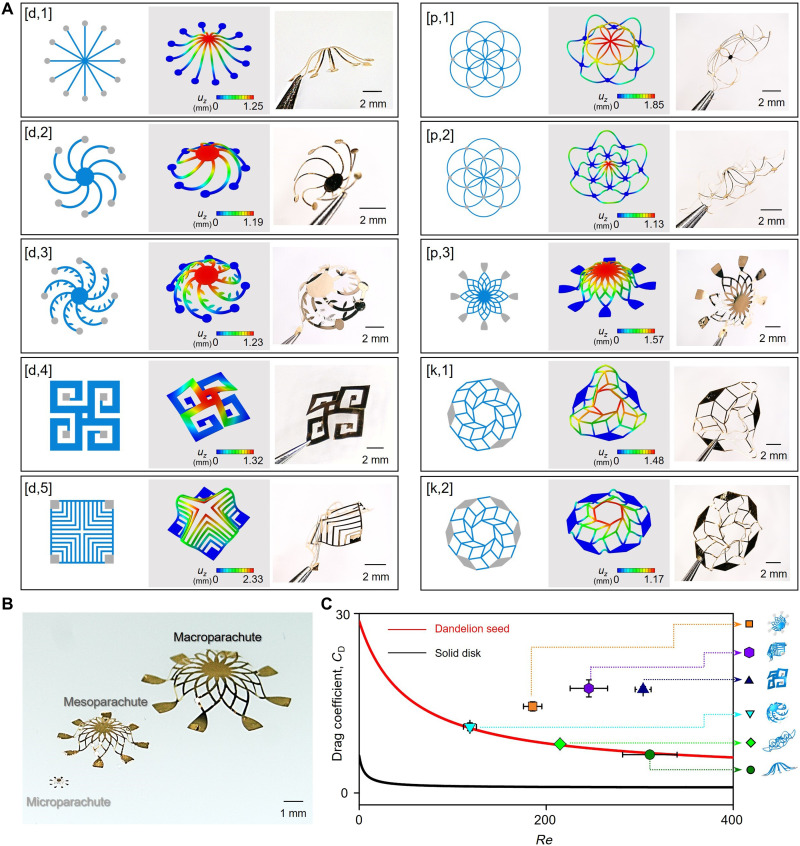
3D fliers with assorted geometries. (**A**) Left: 2D precursors corresponding to dandelion-type structures, [d,1-3]; dome-type structures, [d,4-5]; parachute-type, [p,1-3]; kirigami-inspired type, [k,1-2]. Middle: FEA predictions of 3D flier geometries assembled on elastomer substrates. Right column: Photographs of corresponding free-standing 3D fliers. (**B**) Optical micrograph of a 3D parachute-type flier with micro-, meso-, and macroscale dimensions. (**C**) Plot of the drag coefficient (*C*_D_) for parachute-type fliers as a function of Reynolds number (*Re*). The black and red curves correspond to results for solid disks (*49*) and dandelion seeds (*32*) from previous studies.

Fliers that adopt designs other than those inspired by the *T. australasiae* plant are also of interest. The results in Fig. 3A show layouts that resemble dandelion seeds, domes, parachutes, and abstract kirigami-inspired shapes, each constructed from bilayers of Au/PLGA (~200 nm/60 μm). The simplest 2D precursors (top left in Fig. 3A) with (i) straight branches, (ii) curved hoops, and (iii) lateral protrusions capture certain behaviors of dandelion seeds (*32*). Other examples include precursors that consist of two square shapes designed to transform into 3D dome structures. Different configurations of bonding sites, employed with the previously fabrication process, with a given 2D precursor yield different 3D architectures ([p,1] and [p,2] pair in Fig. 3A). The selection of bonding sites also dictates different 3D kirigami-inspired geometries (bottom right pair). Figure 3B demonstrates parachute-type 3D fliers at sizes from micro- to macroscale. Each of these 10 designs exhibits excellent correspondence between experimentally observed and FEA-predicted shapes (figs. S10 and S11). The levels of prestrain in the PDMS substrate and the overall heights of these 3D fliers scale with the square root of the compressive strain, i.e., ε_max_∝ε_compr_^1/2^, u_z_∝ε_compr_^1/2^. The compressive strain for the 2D to 3D shape transformation relates to the substrate prestrain through ε_compr_ = ε_pre_/(1 + ε_pre_) (*33*).

The Reynolds number, *Re*, characterizes the ratio of inertial to viscous forces associated with flight. For the present systems, *Re* = *UD*/ν, in which *U*, *D*, and ν are the terminal velocity, the diameter of the flier, and kinematic viscosity of air, respectively. The drag coefficient is *C*_D_ = *F*/0.5ρ_a_*U*^2^*A*, where *F* is the drag force, ρ_a_ is the air density, *U*^2^ is the terminal velocity, and *A* is the projected area of the flier. Here, the drag *F* equals the flier weight *W* since, in steady-state falling, the net force in the *z* direction is zero. The terminal velocity *U* follows from drop tests. The projected area *A* is *A* = 0.25π*D*^2^, where *D* is the equivalent diameter. Figure 3C plots the drag coefficient as a function of the Reynolds number for various fliers that adopt dandelion- and parachute-type designs. The results suggest a mechanism for reducing the terminal velocity that is different compared to that of the rotating fliers in Fig. 2. Specifically, the fliers of Fig. 3 create drag to reduce the terminal velocity, while the rotating structures develop lift. The most substantial drag follows from the wall effect near each structural feature (*32*) due to the thick boundary layer that forms at small *Re_d_* (Reynolds number based on the filament thickness), according to $\delta \propto Re_{d}^{-1/2}$ for laminar flow. These parachute-type fliers complement other designs, as they rely on small spacings between filaments to generate wall effects in air flow (*34*). The result is a nonlinear drag coefficient distribution, as shown in Fig. 3C, leading to a large drag coefficient at small *Re* or microscale dimensions. The three dandelion-type fliers lie on a curve fitted from the previously reported experiments on dandelion seeds (*32*), despite the many differences in shapes and levels of porosity.

### Colorimetric assays for pH, UV, and heavy metals

Colorimetric assays are attractive as the basis of “wireless” methods for remote evaluation, via digital image capture and quantitative color extraction (*35*, *36*). Results presented here illustrate use of this simple (*37*), cost-effective means to capture spatiotemporal information on environmental parameters using widely dispersed collections of fliers. As examples, sensing of pH could be important in evaluating the acidity of rainfall (*38*), measurements of UV intensity could be relevant to guiding behaviors to ensure safe skin exposure (*10*), and monitoring of concentrations of heavy metals could be valuable in assessing contaminated ground water (*9*). In all cases, colorimetric reagents integrated onto cellulose substrates via vacuum filtration serve as indicators with high responsivity to targeted parameters, suitable for integration onto the fliers as described previously (*1*). The following reagents are natural compounds (*23*) and/or environmentally benign (*39*, *40*). Details associated with the preparation procedures are in Materials and Methods and fig. S9.

**Fig. 4.**
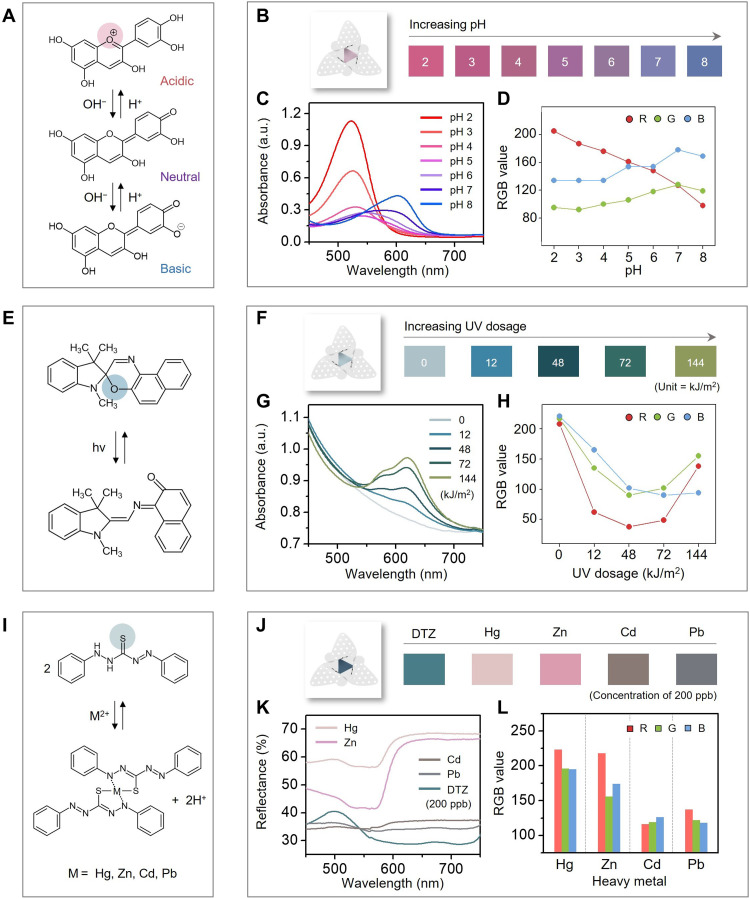
Chemistries and spectral responses of colorimetric assays. (**A**) pH-dependent structural changes in anthocyanin and (**B**) resulting color changes for pH between 2 and 8. Corresponding quantitative analysis conducted by (**C**) UV-visible spectroscopy and (**D**) RGB analysis of digital images. (**E**) UV-dependent structural changes in a photochromic dye (name: 1,3-dihydro-1,3,3-trimethylspiro[2*H*-indole-2,30-[3*H*]naphth[2,1-*b*][1,4]oxazine]) and (**F**) color changes associated with UV dose up to 144 kJ/m^2^. Corresponding quantitative analysis conducted by (**G**) UV-visible spectroscopy and (**H**) RGB analysis of digital images. (**I**) Structural changes in dithizone (DTZ) chelate associated with exposure to heavy metals and (**J**) resulting color changes for different metals, each at 200 ppb. Corresponding quantitative analysis conducted by (**K**) UV-visible spectroscopy and (**L**) RGB analysis of digital images.

The pH assay relies on anthocyanin (*22*), a natural organic compound extracted from red cabbage (Fig. 4A) that exhibits a color response due to interactions with surrounding free hydronium and hydroxyl ions in water (*41*). At a high concentration of hydronium ions (i.e., acidic, pH < 6), the compound generates oxonium ion (O^+^), leading to a red color (*41*). In the relative absence of hydronium ions (i.e., natural, pH 6 to 7; basic, pH >7), the color changes from purple to blue (Fig. 4B). Results of tests indicate that with increasing pH, the absorption peak shifts from a wavelength of 525 to 600 nm, as shown in UV-visible spectra of Fig. 4C. As a result, the RGB values extracted from digital images under white light illumination exhibit a decrease in the red level (Fig. 4D). These color changes are repeatable over 10 cycles of pH between 2 and 8 (fig. S12).

UV sensing follows from the reversible photochromism of spirooxazine. Under UV irradiation, a closed ring in spirooxazine (colorless) changes into an open ring in merocyanine (colored, sea green; Fig. 4E) (*23*). As a result, different colors result from exposure to light in the UV range from 200 to 400 nm. Experiments based on illumination with UV at a wavelength of 365 nm (i.e., UVA) ranging from 0 to 144 kJ/m^2^ (at intensities up to 6 mW/cm^2^; correlated with daily UV index values) (table S1) lead to corresponding color changes (Fig. 4F) due to increased absorbance at 603 nm (Fig. 4G). RGB values extracted from digital images in a range of UV dose are presented (Fig. 4H). For practical cases of relevance here, the color change corresponds approximately to cumulative UV dose (fig. S13).

The colorimetric assay for heavy metals involves a dithizone species as a ligand that binds heavy metal ions to form a sulfur-based complex (Fig. 4I). Dithizone prepared in the form of nanofibers (3.2 ng per indicator) can be exploited to detect heavy metals at concentrations in the parts per billion (ppb) range in water (*9*). Exposure to mercury (Hg), zinc (Zn), cadmium (Cd), and lead (Pb) with a concentration of 200 ppb leads to different changes in color, as determined by reflection mode spectroscopy (Fig. 4K) and by RGB analysis of digital images (Fig. 4L). Although not highly selective, general features of heavy metal pollution in the environment can be ascertained from these color responses (Fig. 4J). Figure S14 demonstrates capabilities for detection across a range of concentrations 2 to 2000 ppb of Hg, Zn, Cd, and Pb. The response time decreases with concentration, e.g., Hg: ~4 hours at 2000 ppb and 24 hours for 2 ppb. The response to Hg is particularly strong due to the high affinity of dithizone for Hg (*42*). In the presence of different species (e.g., Cd or Pb) with Hg, at comparable concentrations, the color change is dominated by the influence of Hg (fig. S15). This type of heavy metal indicator is most relevant for tracking of contamination associated with a known species.

### Practical applications as environmental indicators

The envisioned use of these fliers involves release into the atmosphere such that their passive flight characteristics facilitate wide dispersal into the environment due to interactions with ambient air currents (movie S1). Each flier serves as a sensor of local parameters and/or those associated with its flight trajectory. This scheme complements conventional approaches based on collection of local samples followed by laboratory analysis.

**Fig. 5.**
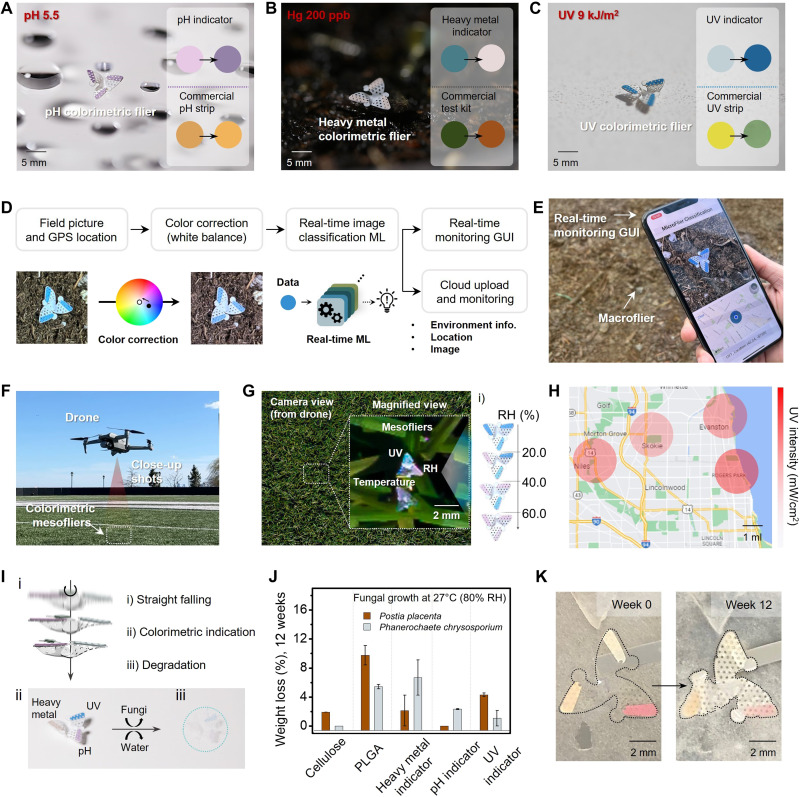
Field tests and fungal degradation studies. Images of a 3D colorimetric mesoflier that responds to (**A**) pH (outdoor rain; pH 5.5), (**B**) heavy metal concentration (200 ppb of Hg solution), and (**C**) UVA exposure (9 kJ/m^**2**^). The insets compare results from the colorimetric assays reported here and from commercial kits. (**D**) Block diagrams of the processes for image-based readout, starting with image capture and image reconstruction based on a color correction algorithm followed by image classification through machine learning (ML). (**E**) A graphical user interface (GUI) on a smartphone captures (i) digital images of a targeted flier, (ii) relevant environment information, and (iii) geographical location. (**F**) Scheme for release of fliers at altitude via a drone. (**G**) Process for collecting digital images of fliers dispersed in this manner using a camera built into the drone [(i) colorimetric humidity assay]. (**H**) Example of environmental mapping or UVA dose using a cloud server. (**I**) Illustration of three stages of the life cycle of a flier, beginning with fabrication and dispersal, responsive colorimetric indication of species of interest, and degradation in the environment driven by hydrolysis and fungus. (**J**) Fungal biodegradation of constituent materials of 3D colorimetric fliers. Percent weight loss for cellulose, PLGA, and colorimetric layers due to degradation via *P. placenta* and *P. chrysosporium* fungi. (**K**) Photographs of a flier after fabrication and after 12 weeks of degradation.

As presented in Fig. 5 (A to C), a set of pilot field studies exploit this mode of use for monitoring (i) rain water (at pH 5.5), (ii) controlled solutions of heavy metals (Hg of 200 ppb), and (iii) solar UV exposure. Separate studies using standard pH strips (pH paper strips, Fisher Scientific), heavy metal detection kits (Heavy Metals Test General Kit, Osumex), a UV digital sensor [Solarmeter model 4.0 (UVA)], and UV strips (UV Fastcheck Strips, UV Process Supply) provide data for comparisons (fig. S16). In all cases, the flier assays produce results that correlate well with these standards, as shown in the insets in Fig. 5 (A to C). Specifically, the pH of rainwater is between 5.0 and 5.5, consistent with some level of acidity (Fig. 5A). As presented in Fig. 5B, the flier for colorimetric assessments of heavy metals yields a value of the concentration of Hg that is consistent with the prepared stock solution and with separate laboratory analysis. The UV indicating flier (Fig. 5C) yields an exposure dose of UVA (13 June 2022, 16:20 to 16:25 at Evanston, IL, USA) that is consistent with measurements determined using the UV digital sensor and with a commercial UV strip. Quantitative analysis involves extraction of RGB values from digital images for all test kits and indicators (fig. S17).

The block diagram in Fig. 5D summarizes an automated scheme that includes steps from data collection to color correction and image classification using a convolutional neural network (CNN) implemented on a smartphone or tablet (*43*). This correction exploits a white balance algorithm based on comparing the color coordinate extracted from the white colorof the flier body to actual white (fig. S18 presenting color correction effect) (*8*). The corrected color of a representative image of a flier appears in Fig. 5D. The next step involves image classification based on training datasets to recognize and label the corrected color into one of several predefined groups (*44*). Images collected from various lighting temperatures, flier orientations and tilt angles, and environmental backgrounds form a training dataset (fig. S19, UV indicators) of 8000 images. The resulting CNN achieves 88.3% classification accuracy computed from the confusion matrix (fig. S20). A graphical user interface (GUI) displays environmental parameters in real time, as well as extracted features from captured images, together with the geographical location determined by Global Positioning System (GPS) (Fig. 5E). A cloud server stores the resulting data (movie S2).

As mentioned previously, drones can be used to release colorimetric fliers into the environment and to capture images of them for evaluation (Fig. 5F and movie S3). Advanced drones offer sophisticated capabilities in geolocation and image acquisition. In the example reported here, a drone operates a 20-MP high-resolution digital camera capable of uploading images to a cloud server for processing. Figure 5G shows colorimetric information on UV dose, as well as humidity (Fig. 5G, i; Humidity Indicators, S-8028) and temperature (Temperature Indicating Label, B-7518) from commercial assays, each extracted from 3D colorimetric mesofliers (~2 mm) deployed in this manner (fig. S21; details for humidity and temperature indicators). This strategy, together with the GPS location information, enables collection and visualization of spatiotemporal properties of an area of the environment, as shown in Fig. 5H (fig. S22 and movie S4). The use of advanced digital cameras and imaging optics can improve on the results presented here.

In these and other applications, the resorbable nature of the fliers is important, as it eliminates the need for recovery (*13*, *20*). Figure 5I illustrates the concept, including dispersal by drone release and passive flight, environmental indication by colorimetric readout, and degradation by natural processes. The envisioned use case is for monitoring environmental status within a short period of time (~1 or 2 days). Multiple cycles of deployment enable monitoring over longer time frames. This last step occurs through the combined action of decomposition by exposure to rain or ground water (hydrolysis) and by bacterial/fungal consumption (decomposition) (Fig. 5I, ii). This latter process can be demonstrated with two different types of decay fungi, brown rot fungus *Postia placenta* and white rot fungus *Phanerochaete chrysosporium*, applied to cellulose, PLGA, and the colorimetric indicators. Here, enzymatic systems of these fungi convert these substances into compost (Fig. 5, J and K) (*13*). Figure 5J presents the average percentage weight loss of these materials after 12 weeks. Each test involves identical samples (*N* = 3) under the same conditions. The PLGA samples show a larger average weight loss (*P. placenta*: 9.54%, *P. chrysosporium*: 4.89%) than the cellulose and indicator samples. For three types of colorimetric indicators, the weight loss is between 0.71 and 4.45%. The rate of degradation of PLGA and cellulose depends on the content of hydrophilic glycolic units,glycolic acid (PGA) for PLGA (*45*) and the nanofibril type, respectively (*13*). Figure 5K shows the decay of a 3D colorimetric flier made of PLGA with three indicators on cellulose substrates due to the action of *P. placenta*, through photographs captured at 0, 4, 8, and 12 weeks (see fig. S23 for 4 and 8 weeks). As shown in Fig. 5K, the fungi partially cover the sample after 4 weeks and fully after 12 weeks.

## DISCUSSION

The environmentally degradable materials, the colorimetric chemical reagents, and the aerodynamic aspects of 3D fliers introduced here represent important advances for this emerging class of large-area, distributed monitoring technology. Specific insights into geometries and mass distributions establish guidelines for achieving optimal flight characteristics with platforms that have diverse, complex geometries designed to support colorimetric assays. Drones as mechanisms for dispersal of these devices and for wirelessly recording their responses offer simple and practical means of application. These concepts are versatile and can be easily extended to other sensing modalities through development of appropriate chemical reagents.

## MATERIALS AND METHODS

### Preparation of colorimetric indicators

Colorimetric indicators for pH, heavy metals, and UV sensing used 0.2 weight % (wt%) of anthocyanin (red cabbage extract; Fluxias GmbH), 100 μl of dithizone solution (2 mM dispersed in acetone; Sigma-Aldrich), and 0.5 wt% of spirooxazine (1,3-dihydro-1,3,3-trimethylspiro[2*H*-indole-2,3′-[3*H*]naphtha[2,1-*b*][1,4]oxazine; Sigma-Aldrich) dissolved into 100 ml of deionized (DI) water, 30 ml of water (buffered to 2; Fisher Scientific), and 20 ml of a mixture of acetone and DI water (1:1 of volume ratio), respectively (*9*). Vacuum filtration of these solutions through mixed cellulose ester membranes (47 mm diameter, 80 μm thick, 0.1 μm pore; MF-Millipore) formed colorimetric assays. Laser cutting processes yielded individual indicators in shapes to allow mounting on fliers (fig. S9).

### Bioresorbable, 3D micro-, meso-, and macrofliers

Fabrication of 2D precursors in PLGA began with laser ablation to define the desired shapes from uniform thin films (thickness of ~60 μm). Perforation for PLGA and colorimetric assays occurs during the laser ablation process. Transfer onto a PDMS substrate and bonding of cellulose-supported colorimetric assays at targeted locations represented the next step in the fabrication. Applying pressure with a piece of PDMS at elevated temperatures (95°C, 15 min) softened the PLGA to facilitating bonding with the cellulose, without the need for a separate adhesive. The next step involved transferring the 2D PLGA/colorimetric precursors onto a prestretched silicone elastomer substrate (Dragon Skin, Smooth-On) with small blocks of PDMS located at anchor sites. Releasing the prestrain led to mechanical buckling and a corresponding 2D to 3D geometric transformation. Heating to 70°C for 1 min in an oven relaxed the strains in the PLGA (glass transition of 50° to 55°C) such that cooling to room temperature fixed the 3D shape. Stretching PDMS again released free-standing 3D objects. This scheme applies equally well at large scales, with structures that have dimensions in the range of centimeters.

### Wake dynamics of free-falling fliers via PIV

Results of two PIV experiments allowed comparisons of the wake dynamics of free falling of two types of fliers, case1 and case3. In each experiment, the fliers fell from a height of 96 cm in the center of an acrylic box with a 20 cm × 20 cm square cross-section. Oil droplets introduced into the box served as tracer particles. The PIV system included an Nd:YLF laser with pulse energies of 50 mJ (527-80-M, Terra), with a sample frequency of 300 Hz, equivalent to Δ*t* = 3.33 ms, a digital camera (2560 pixels × 1600 pixels, CMOS Phantom Miro 340), a synchronizer (LaserPulse Synchronizer Model 610036, TSI), and a PIV control software (TSI Inc.). Optical components shaped the output of the laser into the geometry of a sheet to define an illuminating plane through which each flier passed during free fall. For each experiment, the camera captured 1000 frames at a sampling frequency of 300 Hz. The field of view covered a 60 mm × 100 mm region located 150 mm above from the bottom of the box. These image sequences were interrogated using a direct Fourier transform correlation via PIVlab (*46*). The final interrogation window had a size of 16 pixels × 16 pixels with 50% overlap, resulting in a vector grid spacing of 0.606 mm along both *x* and *y* axes.

### Fungal biodegradation tests of colorimetric 3D flier

The fungal degradation test involved two species, *P. placenta* (Fr.) M.Lars. and Lomb. (MAD 698) and *P. chrysosporium* (ME461), obtained from the Forest Products Laboratory, Madison, Wisconsin. Growth occurred on 2% malt agar, at 27°C and 80% relative humidity (RH) for 3 to 4 weeks before introduction of the test samples, according to American Society for Testing Materials (ASTM) Standard D4445-91 (ASTM 1998) (*47*). Placing five groups of films (cellulose, PLGA, pH colorimetric cellulose, heavy metal colorimetric cellulose, and UV colorimetric cellulose) in petri dishes containing fungal growth allowed for continued incubation at 27°C and 80% RH for 12 weeks. Photographs collected on weeks 0, 4, 8, and 12 served as measures of growth and degradation. Guidelines of American Wood Protection Association (AWPA) Standard E10-12 (AWPA 2014), with some modifications (*48*), dictated conditioning of the test samples and measurements of their original weights before their placement on malt agar plates containing a confluent growth of fungal mycelia. Removal of fungal mycelia occurred on the 12th week. Weight measurements occurred again after low heat drying of the sample set on a rack for 2 days followed by reconditioning at 27°C and 80% RH for 4 days. The results included the average percentage weight loss computed from these measured weights.

### Finite element analysis

3D FEA performed with the commercial software package Abaqus revealed the nonlinear postbuckling behaviors of the 2D precursor structures. The results included the deformed 3D configurations and strain distributions at different levels of compression associated with relaxing the prestretched substrate. Eight-node solid elements defined the mesh for both this substrate and the 2D precursor structures. Tests of convergence of the mesh size ensured computational accuracy. In the simulations, the Mooney-Rivlin strain energy potential model captured the hyperelastic behavior of the elastomer substrates. Dragon Skin 10 was modeled as incompressible, with an elastic modulus *E*_DragonSkin_ = 166 kPa. PLGA and cellulose ester were modeled as linear elastic materials, with the elastic modulus and Poisson’s ratio given by *E*_PLGA_ = 1.37 GPa and ν_PLGA_ = 0.44 for PLGA and *E*_cellulose_ = 340 MPa and ν_cellulose_ = 0.4 for cellulose ester. Gold (Au) was modeled as an idealized elastoplastic material (without hardening; yield strain chosen as 0.3%), with Young’s modulus and Poisson’s ratio of *E*_Au_ = 78 GPa and ν_Au_ = 0.44.

## Supplementary Materials

### This PDF file includes:

Notes S1 and S2 Table S1 Figs. S1 to S23 References
