# Supplementary material for: Biodegradable, three-dimensional colorimetric fliers for environmental monitoring
Source: Sci Adv. 2022 Dec 23;8(51):eade3201. doi: 10.1126/sciadv.ade3201 (PMC9788784; doi:10.1126/sciadv.ade3201)
Supplement: Supplementary file 1 — Notes S1 and S2 Table S1 Figs. S1 to S23 References [file sciadv.ade3201_sm.pdf]

Science Advances
AAAS

Supplementary Materials for

Biodegradable, three-dimensional colorimetric fliers for environmental monitoring

Hong-Joon Yoon *et al.*

Corresponding author: Leonardo P. Chamorro, lpchamo@illinois.edu; Yoonseok Park, yoonseok.park@khu.ac.kr; John A. Rogers, jrogers@northwestern.edu

Sci. Adv. **8**, eade3201 (2022)
DOI: 10.1126/sciadv.ade3201

The PDF file includes:

Notes S1 and S2
Table S1
Figs. S1 to S23
Legends for movies S1 to S4
References

Other Supplementary Material for this manuscript includes the following:

Movies S1 to S4

Supplementary Note 1:
Terminal and rotational velocities

1. **Equations of motion.**

Let's consider a global coordinate to describe the microflier motion. A coordinate system *XYZ* is fixed to the earth that follows the standard right-hand rule direction, with Z pointing against gravity (**Fig. S4**).

The aerodynamic of the microflier may undergo complex fluid-structure feedback even without auto-rotation. Auto-rotation affects coherent motions and generates leading-edge vortices that contribute to lift production; the combined processes challenge modeling. Lift and drag of the flier depend strongly on the flow, fluid properties, namely, fluid density, ρ_f, kinematic viscosity, ν, and material properties including density, ρ_m, geometry (coning angle, *β*, wing area, wing aspect ratio, among others) and flight status (falling speed, *u*, rotational velocity, *ω*, advance ratio, and others):

$$(v_T, \omega_T) = f\begin{pmatrix}\text{fluid, flow and flier properties}\\ \text{flier geometry}\\ \text{flight status}\end{pmatrix}$$

Here, we represent the microflier with three flat-plate wings of varying chord lengths, $c = c(r)$, where r is the radial distance. The blades have a coning angle, $\beta = 22°$, and the airflow velocity is U, and the effective angle of attack is α, both functions of the wing radial position, $U = U(r)$ and $\alpha_E = \alpha_E(r)$, where U and α_E are defined as follows

$$U = \sqrt{v_T^2 + (\omega_T r)^2}$$

$$\alpha = \alpha_R + \tan^{-1}(\frac{\omega_T r}{v_T})$$

where $\alpha_R(r)$ is the local pitch angle of the flier wing. Considering the linear and angular momentum balances for the linear and rotational acceleration about the z axis, the governing equations of motion are given by:

$$m\dot{u}_z = \int_0^R \tfrac{1}{2}\rho_f({v_T}^2 + {\omega_T}^2 r^2)c(r)[C_L(r)\cos\alpha + C_D(r)\sin\alpha]\cos\beta\, dr - mg$$

$$I_z\ddot{\Phi}_z = \int_0^R \tfrac{1}{2}\rho_f({v_T}^2 + {\omega_T}^2 r^2)c(r)[C_L(r)\sin\alpha + C_D(r)\cos\alpha]\cos\beta r\, dr$$

For the steady falling stage, these equations reduce to total lift balancing the microflier weight and zero net torque.

2. Lift and drag estimation.

Lift and drag forces are needed for robust estimation of the terminal vertical velocity, v_T, and terminal rotational velocity, ω_T. Leading-edge vortices, which increase lift performance and delay the stall for autorotating fliers (*50, 51*), are accounted with the so-called suction model proposed (*52*). It provides an estimation of the aerodynamic lift; the normal force is then used for drag force estimation, as follows:

$$C_L = C_{L\alpha,3D}\sin\alpha\cos\alpha\, K_{pol}(\alpha)$$

$$C_D = C_L\tan\alpha$$

where, $C_{L\alpha,3D}$ is the slope of the wing lift versus angle of attack α. It can be estimated using the extended lifting line theory (*53*) for low aspect ratio (AR < 3) wings; it gives $C_{L\alpha,3D} = \frac{C_{L\alpha,2D}}{E + kC_{L\alpha,2D}/(\pi AR)}$. Here, $C_{L\alpha,2D} = 2\pi$ for flat plates, $E \approx 1$ is the edge correction factor, $k \approx$

1.37 accounts for the downwash distribution of the wings (*51,54-55*). The corresponding drag and lift coefficients are given in **Fig. S6**.

3. **Solution – iterative procedure.**

The lift and drag equations applied in the complex geometry of the microflier wing, analytic solutions for v_T and ω_T are not available. An arbitrary initial value v_T allows a corresponding ω_T using the torque equation (**Eqn. S3**) and then the effective angle of attack, α_E, and total velocity U following **Eqn. S2**; then the linear momentum balance in **Eqn. S3** is re-evaluated to update v_T. This process continues until the force and torque equations in **Eqn. S3** are satisfied; see the flow chart illustrating the iterative process in **Fig. S7**.

Supplementary Note 2:
Stability analysis

The modeling of the microflier flight behavior requires a balance between the aerodynamic loads and the microflier weight and inertia. In addition, to satisfy the equations of motion, stability is also necessary for a steady falling pattern.

4. **Euler equation – rotation.**

We start from the general Euler's rotation equation set that describes rigid body rotation as follows

$$I_1\dot{\omega}_1 + (I_3 - I_2)\omega_2\omega_3 = T_1$$

$$I_2\dot{\omega}_2 + (I_1 - I_3)\omega_3\omega_1 = T_2$$

$$I_3\dot{\omega}_3 + (I_2 - I_1)\omega_1\omega_2 = T_3$$

Here, T_1, T_2 and T_3 are the torque with respect to *X*, *Y* and *Z* axes. The microflier can then be treated as an oblate symmetric top plate, where there are only nonzero diagonal terms, I_{11}, I_{22} and

I_{33} within the moment of inertia matrix and $I_{33} > I_{22} = I_{11}$. This results in $\omega_3 = C$, where C is a constant equal to the initial rotation speed upon release, i.e., zero in our experiments. This suggests that the pitching stability is crucial for the onset of autorotation and zigzag noted in case (i).

5. Pitch stability.

Several parameters affect the pitch stability, i.e., rotation about x-axis, in the simplified three-flat-plate wing microflier geometry. First, the pitch angle, φ, is positive for counter-clockwise rotation about the x-axis, and the incidence angle, γ, is defined as that between the flow direction and the flat plate wing chord line.

Here, the aerodynamic forces on the wing are represented as one resultant force acting through the center of pressure CP; similarly, the microflier weight acts at the center of gravity CG. Additionally, Δz is the height of the center of gravity location taken the 'circular' base plate as the datum; see schematic in **Fig. S5** for clarity. The characteristic lengths r_{p1}, r_{p2}, r_{p3}, and r_m are the distance between x−axis and CP_1, CP_2, CP_3 and CG.

The total pitching torque, T_x, can be obtained by accounting for the contributions from the aerodynamic forces from the product of the three wings and the microflier weight with the respective moment arm. Note that it is possible to select a position with the third wing aligned with the x-axis with r_{p3}; thus, T_x is simplified to

$$T_x = L_2 r_{p2} - L_1 r_{p1} - mg r_m$$

Since r_p increases as incidence angle γ increases (*56*), it is possible that the net torque created by aerodynamic forces between wings, i.e., $L_2 r_{p2} - L_1 r_{p1}$, counterbalances the torque generated from

weight, i.e, $mg \times r_m$. To satisfy this criterion, the incidence angle, γ, must be large enough so that it is sufficiently negative to enable the stable condition

$$\frac{dT_x}{d\varphi}|_{\varphi_0} < 0$$

where $\varphi_0 = 0$ in our case is the equilibrium pitch angle. A stable configuration is provided in **Fig. S8** neglecting the wing that do not contribute to the torque, T_x, to illustrate the stabilizing mechanism.

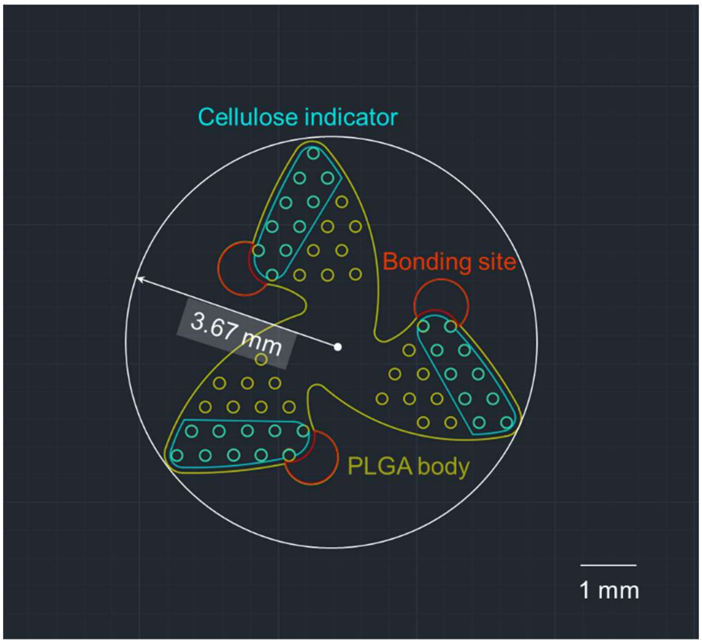

Fig. S1. Basic simplified design schematic of the rotating mesoflier for theoretical analysis.

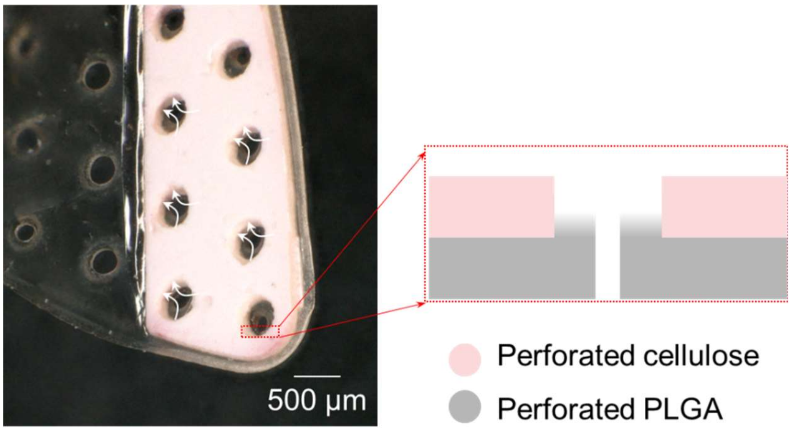

Fig. S2. Expanded view of a contact bonding between perforated PLGA and perforated cellulose. After thermal treatment at temperature slightly above the glass transition of PLGA, perforated part is transformed into the perforated side on the cellulose side.

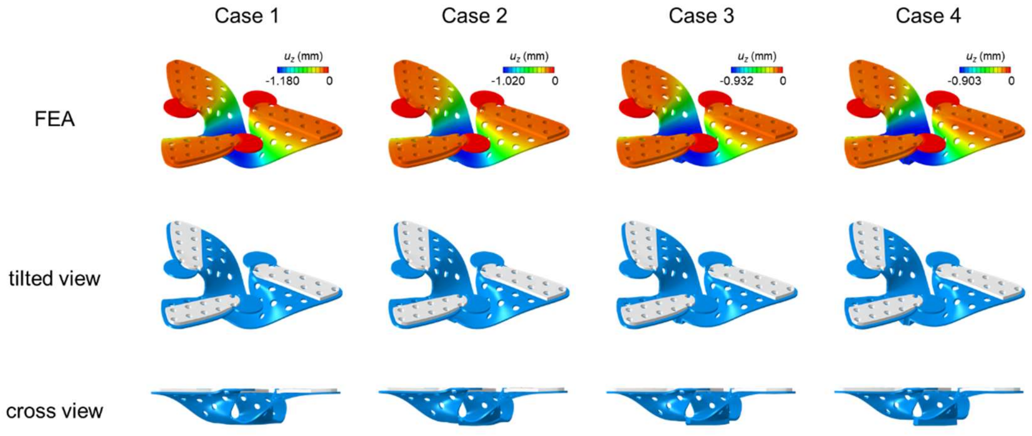

Case	Mass (mg)	Center of mass (mm)
**1**	1.22	-3.71e-005, -4.22e-006, -0.298
**2**	1.28	-4.27e-005, -1.54e-005, -0.336
**3**	1.34	2.09e-006, -6.19e-005, -0.350
**4**	1.41	9.17e-006, -9.28e-005, -0.378

Fig. S3. Computed center of mass of fliers as a function of the mass as an anchor material. For case 1, there is no anchor material. Case 2-4 gain masses, respectively, as shown in table.

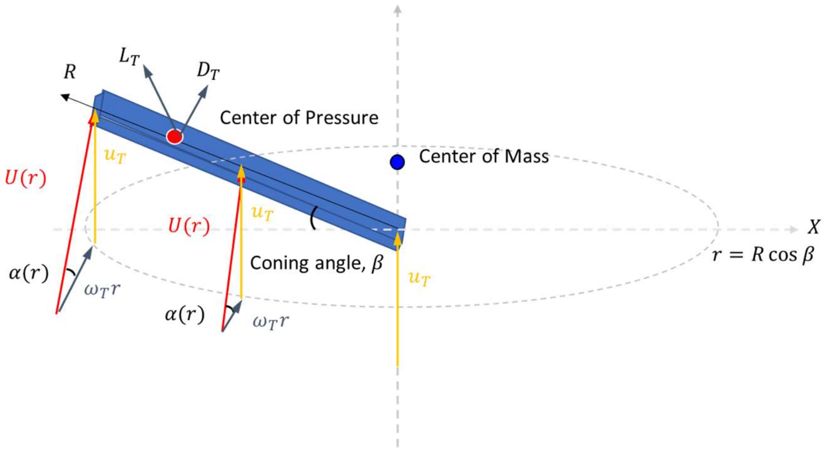

Fig. S4. Basic simplified model schematic of the rotating flier for theoretical analysis.

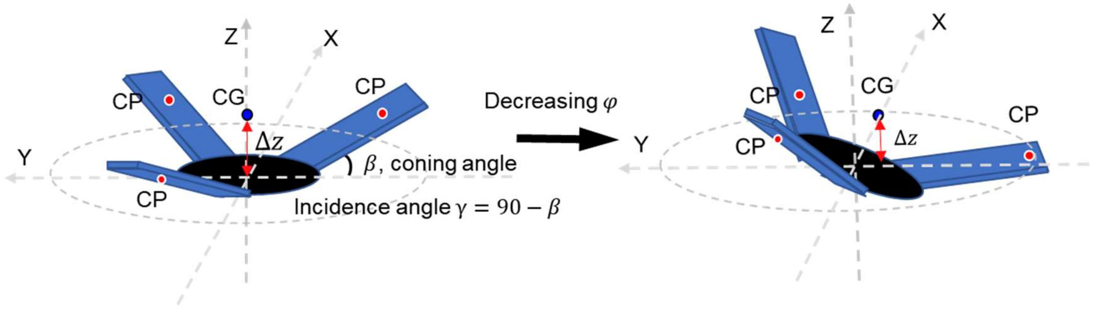

Fig. S5. Schematic diagram of a rotating microflier.

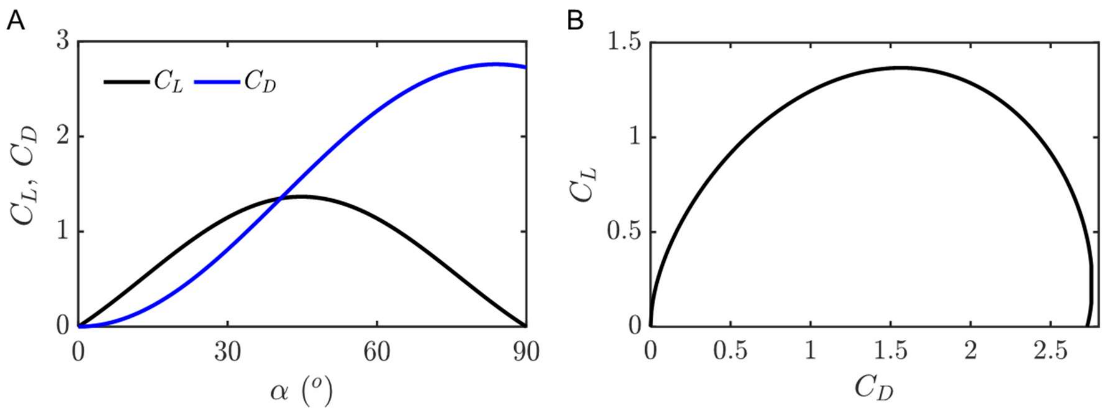

Fig. S6. Flier lift and drag. (A) Lift (black) and drag (blue) coefficient as a function of angle of attack. (B) lift coefficient versus drag coefficient.

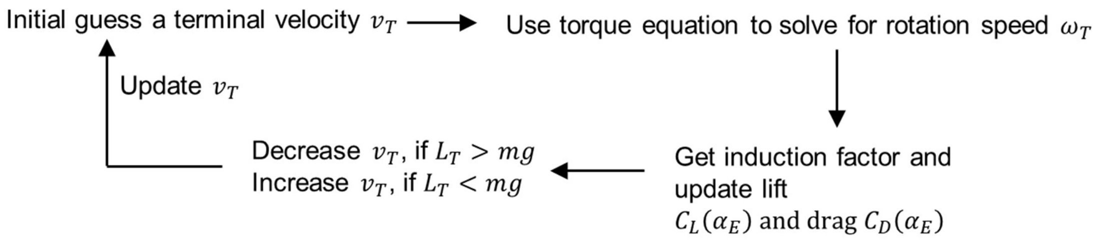

Fig. S7. Flow chart of the iterative procedure adopted to solve for terminal velocity and terminal rotation speed.

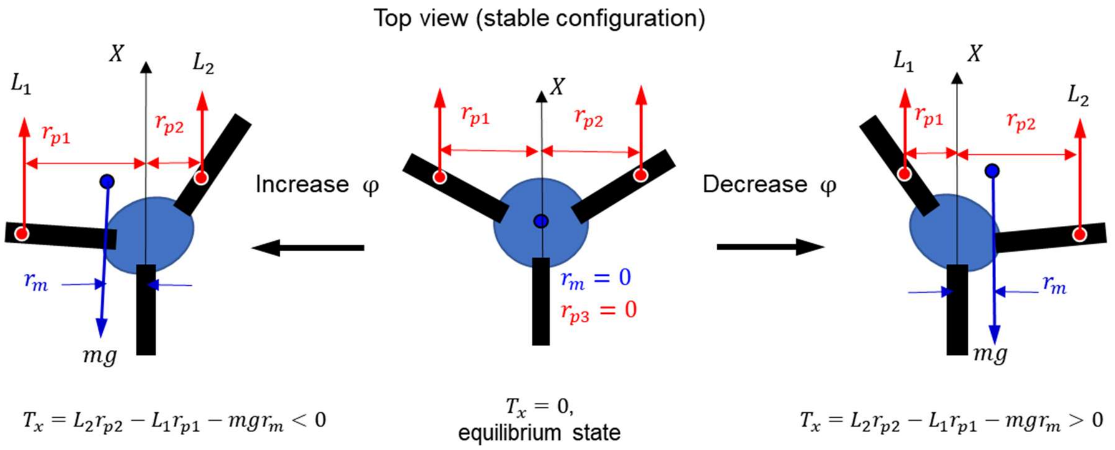

Fig. S8. top view of the stable pitching configuration demonstrating the stability criteria $\frac{dT_x}{d\varphi}|_{\varphi=0} < 0$

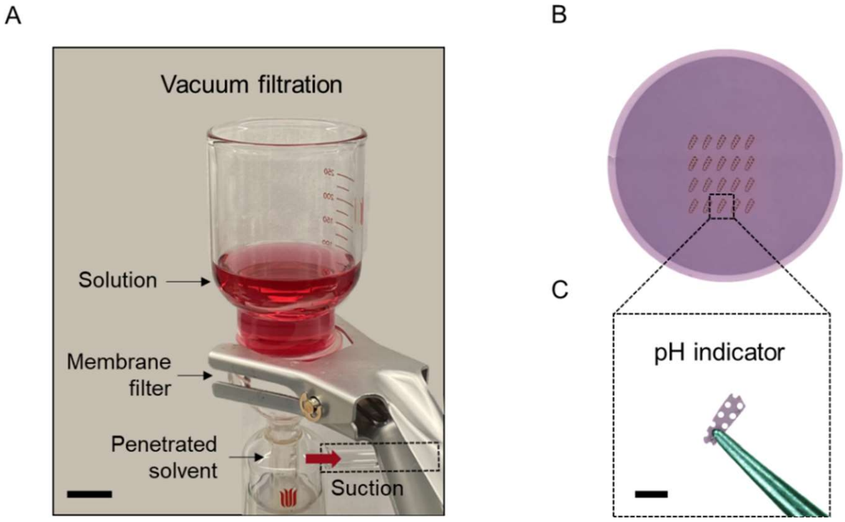

Figure S9. Details associated with preparation procedures in colorimetric assays.
(**A**) Experimental setup for vacuum filtration. Scale bar, 2 cm. (**B**) Colorimetric assay for pH sensing. (**C**) Laser-cut membrane as an individual indicator for mounting on a flier. Scale bar, 1 mm.

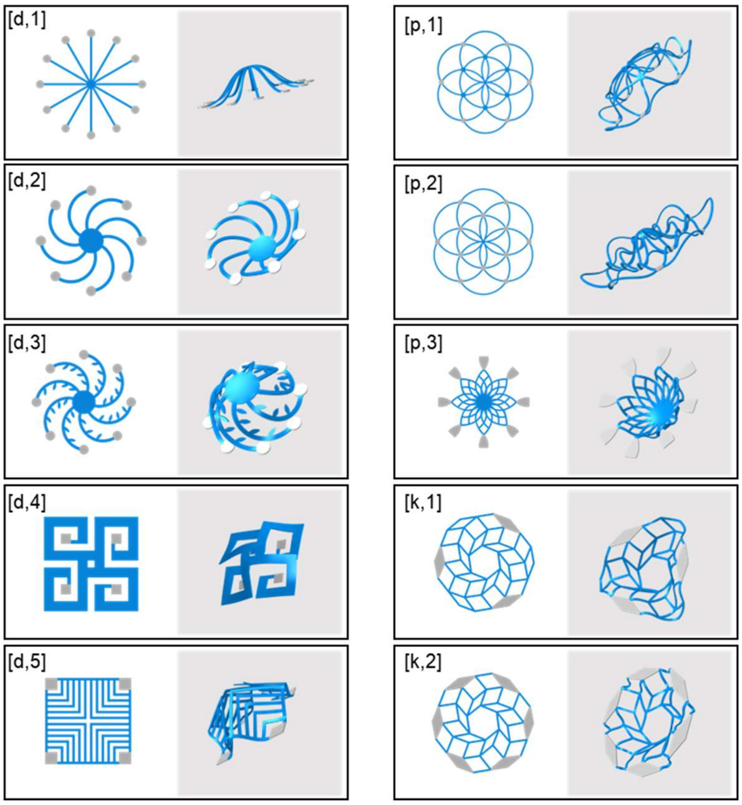

Fig. S10. Computed diverse buckled, free-standing 3D structures inspired by dandelion seeds, domes, parachutes, and kirigami shape. The FEA results match experimental observations in Fig. 3A. 2D precursors shown on each column left are transformed into 3D architectures.

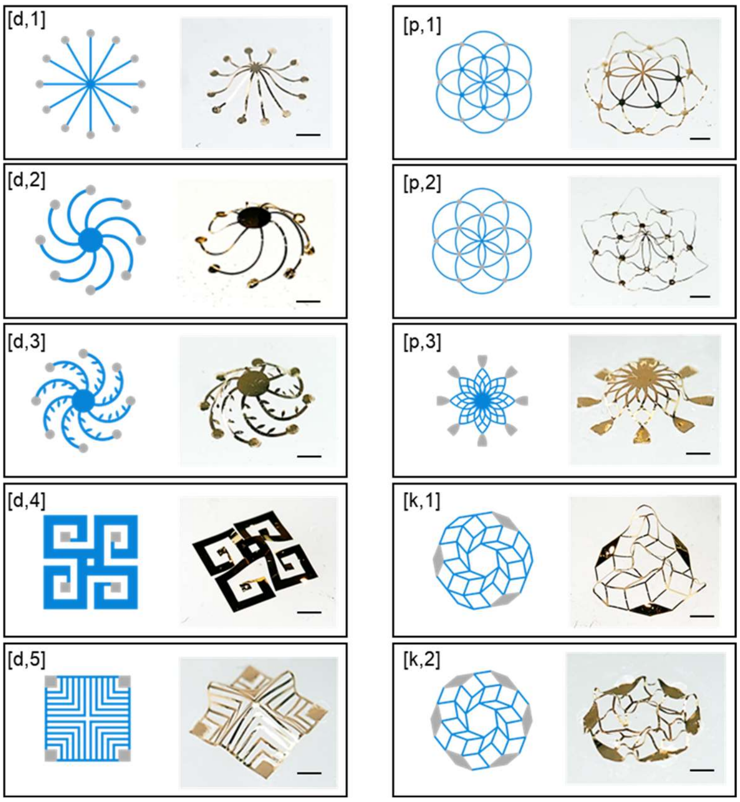

Fig. S11. Experimentally transformed diverse 3D structures inspired by dandelion seeds, domes, parachutes, and kirigami shapes. The FEA and experimental results are in strong agreement, as in Fig. 3A. 2D precursors shown on each column left are mechanically buckled into 3D architectures (photo images on each column right) (scale bar: 2 mm).

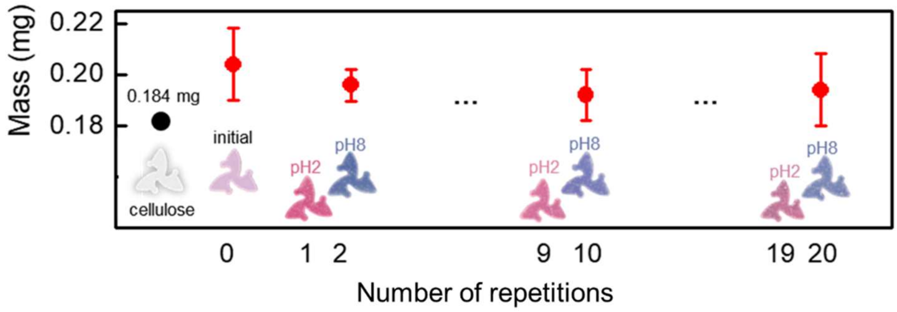

Fig. S12. Repetitivity of colorimetric pH indicator. Repetitivity is experimentally verified as observing color change upon exposure of the indicator to pH 2 and 8 up to ten cycles.

**Date (yyyy/mm/dd)**	**Time**	**UV index**	**UV intensity (unit: mW/cm^2)**
**2022/06/13**	12:45	9	4.4
	13:45	9	4.3
	15:10	7	3.6
	16:25	5	3.1
	17:20	3	2.2
	18:30	1	1.1
**2022/06/16**	13:32	9	4.5
	14:25	8	4.1
	15:20	7	3.8
	16:20	5	3.4
	17:15	3	2.9
	18:10	1	1.7

Table S1. **UV intensities at various times, corresponding to UV index**

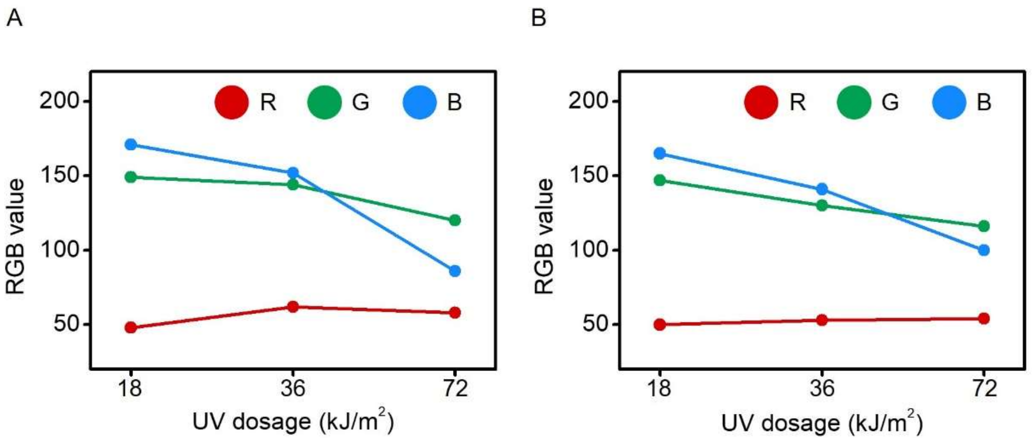

Fig. S13. RGB values of colorimetric UV indicators under varying UV dosages. (A) Results measured at 2 mW/cm^2 of UV intensity for 15, 30, 60 minutes. (B) Results measured at 6 mW/cm^2 of UV intensity for 5, 10, 20 minutes.

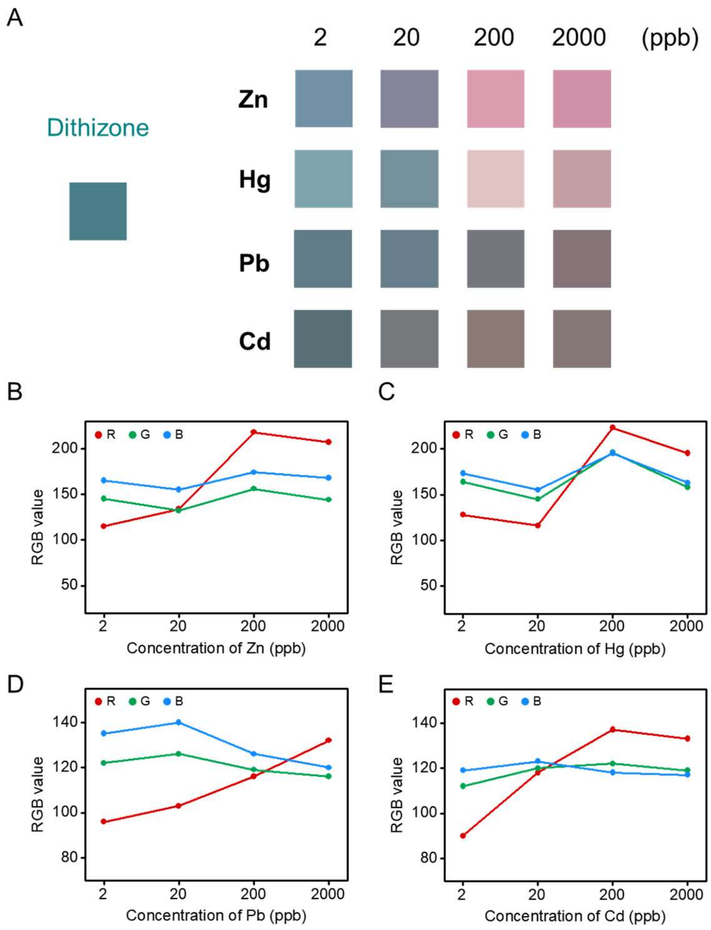

Fig. S14. Quantitative colorimetric analysis of the responses of the heavy metal assays. (A) optical images of colorimetric indicators at various concentrations of Zn, Hg, Pb, Cd ranging from 2 to 2000 ppb. RGB values of the images from (B) zinc, (C) mercury, (D) lead, (E) cadmium.

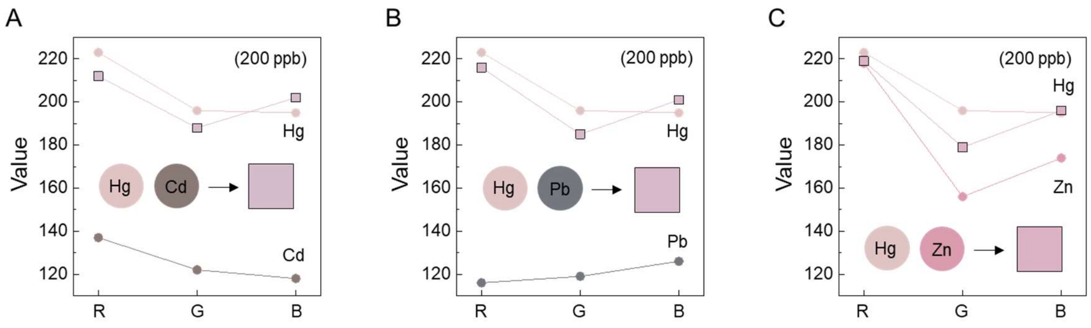

Fig. S15. Quantitative colorimetric analysis in selective extraction of heavy metals. RGB values extracted from colorimetric indicators following immersion in mixtures with different species of heavy metal ions, (**A**) Hg and Cd; (**B**) Hg and Pb; and (**C**) Hg and Zn.

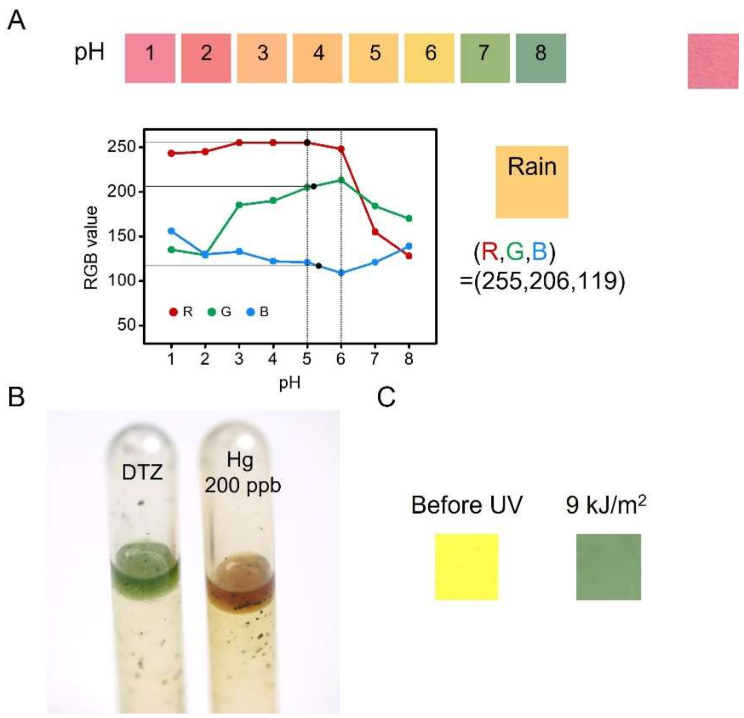

Fig. S16. Commercial colorimetric assays for (A) pH and response to rain water, (B) heavy metal and response to Hg, (C) UV and response to UVA 9 kJ/m^2.

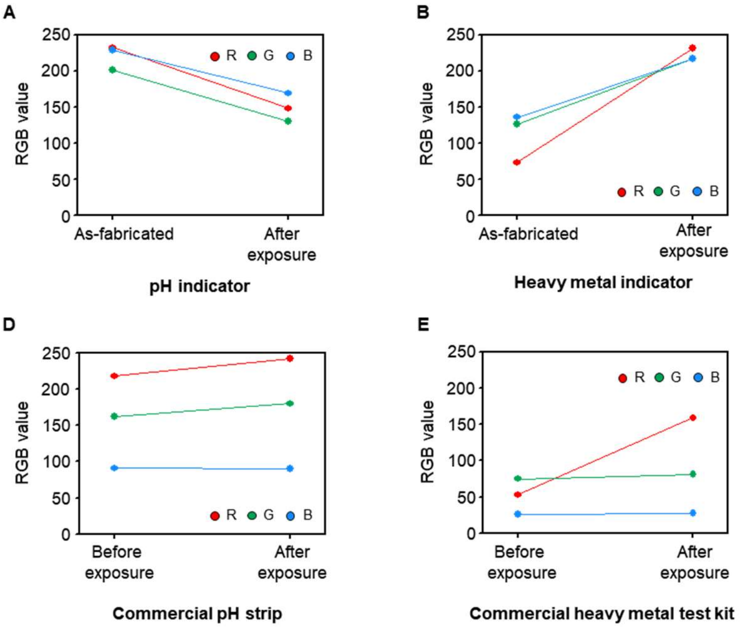

Fig. S17. Quantitative colorimetric analysis in (A-C) as-fabricated, and (D-E) commercial indicators.

RGB values extracted from colorimetric indicators exposed to i) acid rain (A, D), ii) heavy metal (Hg) (B, D), and iii) UV (C, F).

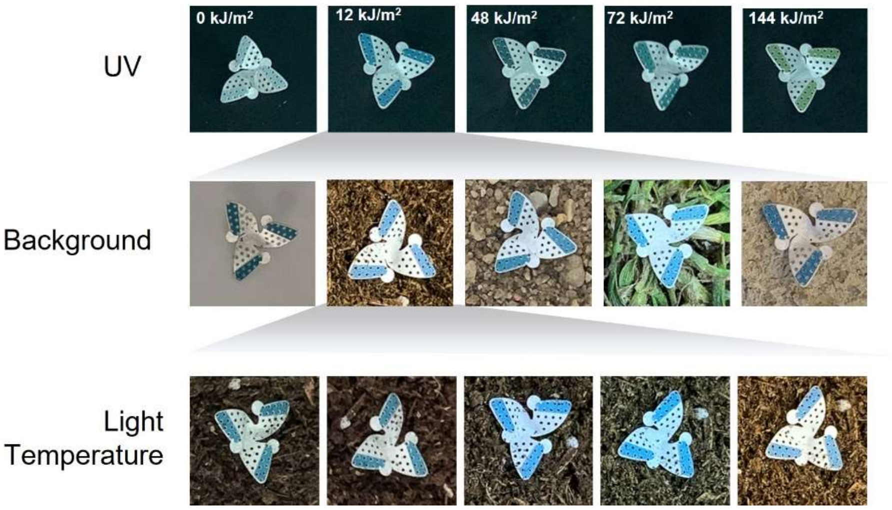

Fig. S18. Colorimetric flier images under various light temperatures and backgrounds for training data set

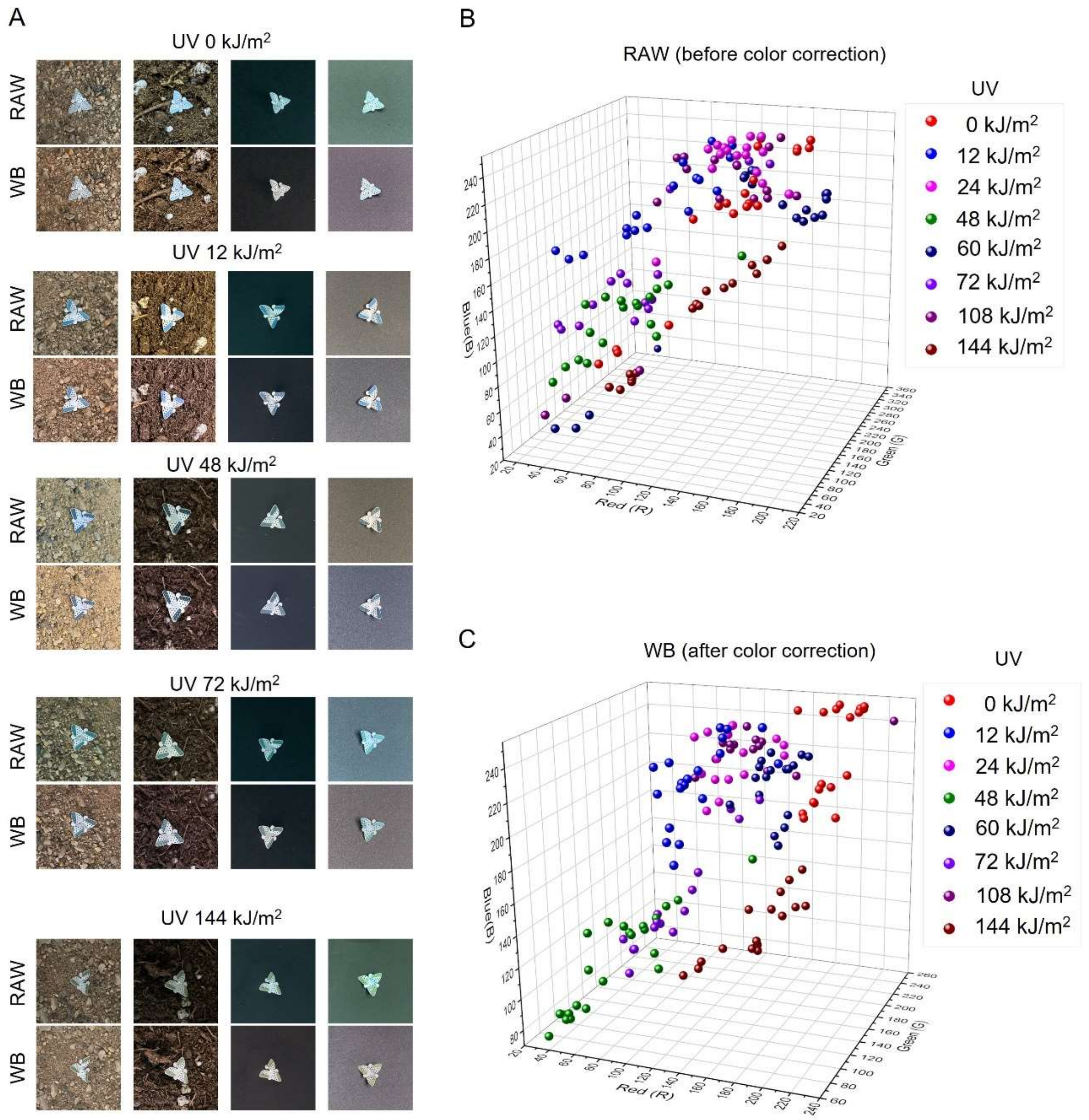

Fig. S19. Color correction results from the diverse environment. (A) Raw and color corrected image of colorimetric fliers under the various UV dosages. RGB color distribution of colorimetric fliers in the (B) raw images and (C) color corrected images.

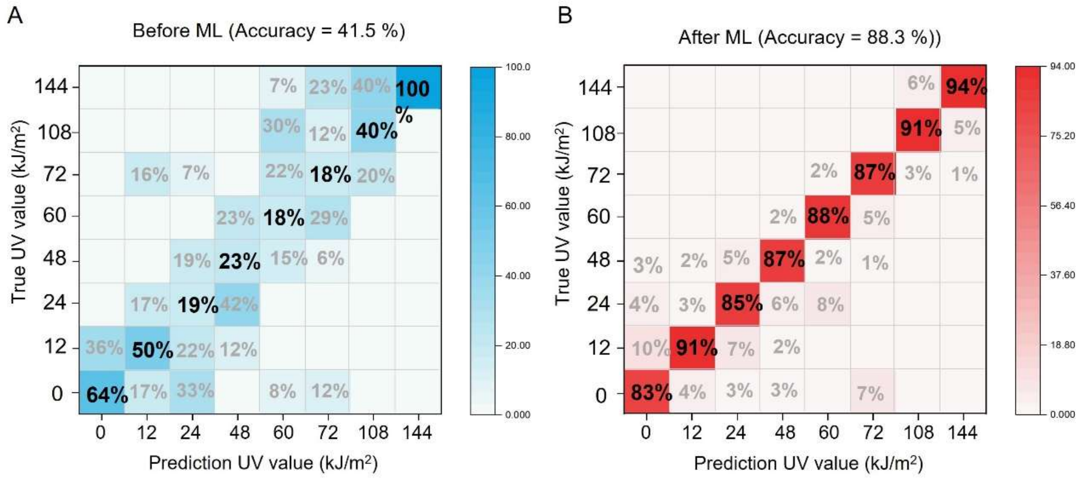

Fig. S20. Confusion matrix (8,000 training images, 2,000 test images) for UV classification of the colorimetric flier (A) before and (B) after machine learning process.

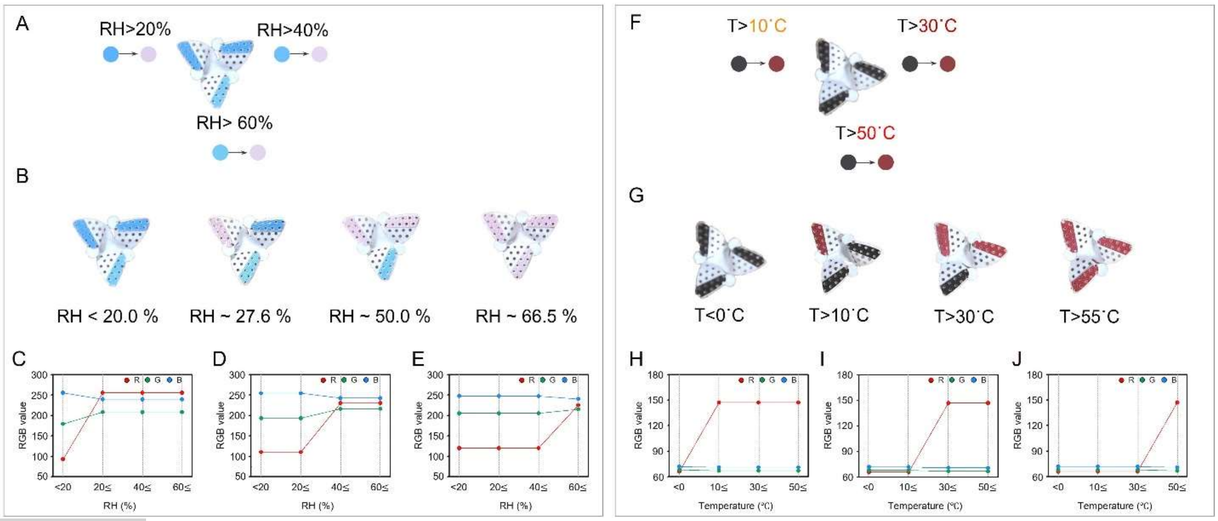

Figure S21. Colorimetric indicators for measuring relative humidity (RH) and temperature. **(A)** Resulting color changes associated with (**B**) different RH conditions, and **(C to E; RH>20%, RH>40%, and RH>60%)** corresponding quantitative analysis conducted by RGB analysis of digital images, respectively. **(F)** Resulting color changes for temperature ranging from (**G**) 0 to 55℃, and **(H to J; T>10**℃, **T>30**℃, and **T>50**℃**)** corresponding quantitative analysis conducted by RGB analysis of digital images, respectively.

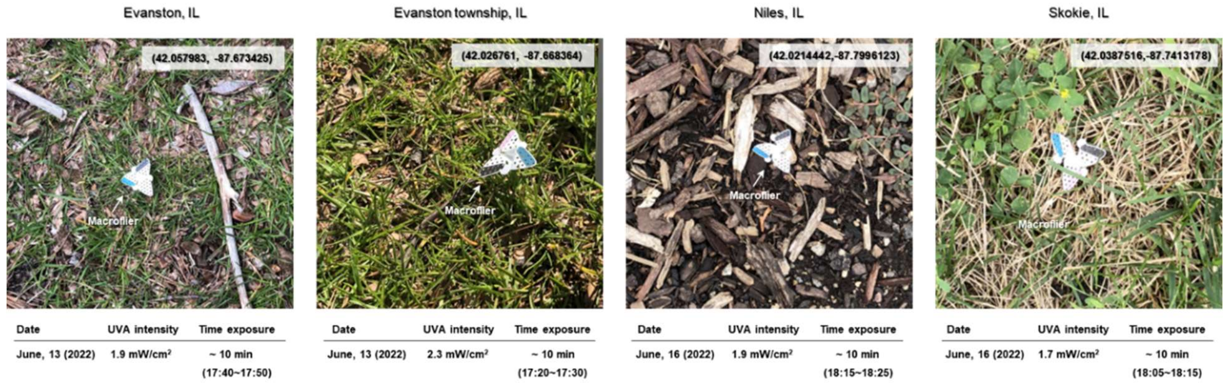

Fig. S22. Captured images of deployed colorimetric fliers at different locations to visualize spatio-temporal environmental status (here, UV dose).

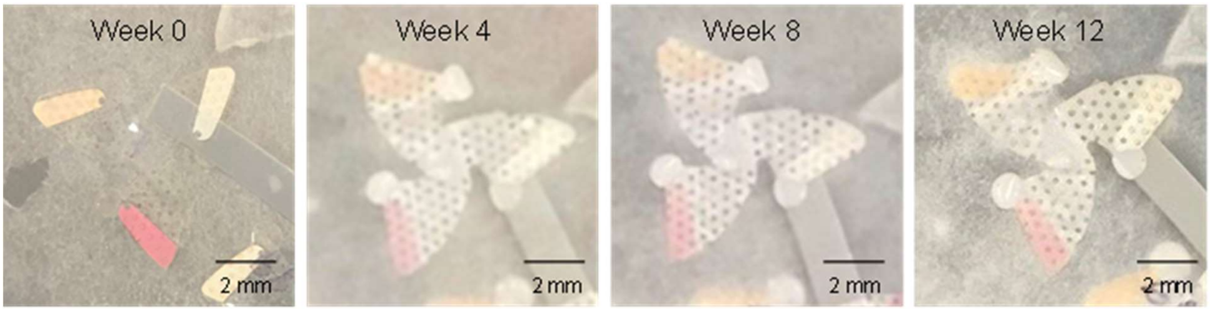

Fig. S23. Magnified photographs of the colorimetric flier during the degradation process. The fungus gradually covers the filer.

Video S1 | Four types of falling behaviors depending on different anchor materials

Video S2 | Real-time environmental UV monitoring GUI (left video) and environmental geographic map in the cloud server (right video)

Video S3 | Spreading of the colorimetric fliers using a drone system

Video S4 | Environmental monitoring using drone system (left video), video of drone camera and the geographic map in the cloud server (right video)

REFERENCES AND NOTES

1. B. H. Kim, K. Li, J.-T. Kim, Y. Park, H. Jang, X. Wang, Z. Xie, S. M. Won, H.-J. Yoon, G. Lee, W. J. Jang, K. H. Lee, T. S. Chung, Y. H. Jung, S. Y. Heo, Y. Lee, J. Kim, T. Cai, Y. Kim, P. Prasopsukh, Y. Yu, X. Yu, R. Avila, H. Luan, H. Song, F. Zhu, Y. Zhao, L. Chen, S. H. Han, J. Kim, S. J. Oh, H. Lee, C. H. Lee, Y. Huang, L. P. Chamorro, Y. Zhang, J. A. Rogers, Three-dimensional electronic microfliers inspired by wind-dispersed seeds. *Nature* **597**, 503–510 (2021).

2. H. Luan, Q. Zhang, T.-L. Liu, X. Wang, S. Zhao, H. Wang, S. Yao, Y. Xue, J. W. Kwak, W. Bai, Y. Xu, M. Han, K. Li, Z. Li, X. Ni, J. Ye, D. Choi, Q. Yang, J.-H. Kim, S. Li, S. Chen, C. Wu, J.-K. Chang, Z. Xie, Y. Huang, J. A. Rogers, Complex 3D microfluidic architectures formed by mechanically guided compressive buckling. *Sci. Adv.* **7**, eabj3686 (2021).

3. Y. Park, C. K. Franz, H. Ryu, H. Luan, K. Y. Cotton, J. U. Kim, T. S. Chung, S. Zhao, A. Vazquez-Guardado, D. S. Yang, K. Li, R. Avila, J. K. Phillips, M. J. Quezada, H. Jang, S. S. Kwak, S. Min Won, K. Kwon, H. Jeong, A. J. Bandodkar, M. Han, H. Zhao, G. R. Osher, H. Wang, K. H. Lee, Y. Zhang, Y. Huang, J. D. Finan, J. A. Rogers, Three-dimensional, multifunctional neural interfaces for cortical spheroids and engineered assembloids. *Sci. Adv.* **7**, eabf9153 (2021).

4. M. Han, X. Guo, X. Chen, C. Liang, H. Zhao, Q. Zhang, W. Bai, F. Zhang, H. Wei, C. Wu, Q. Cui, S. Yao, B. Sun, Y. Yang, Q. Yang, Y. Ma, Z. Xue, J.W. Kwak, T. Jin, Q. Tu, E. Song, Z. Tian, Y. Mei, D. Fang, H. Zhang, Y. Huang, Y. Zhang, J. A. Rogers, Submillimeter-scale multimaterial terrestrial robots. *Sci. Robot.* **7**, eabn0602 (2022).

5. J. Rabault, R. A. Fauli, A. Carlson, Curving to fly: Synthetic adaptation unveils optimal flight performance of whirling fruits. *Phys. Rev. Lett.* **122**, 024501 (2019).

6. R. A. Fauli, J. Rabault, A. Carlson, Effect of wing fold angles on the terminal descent velocity of double-winged autorotating seeds, fruits, and other diaspores. *Phys. Rev. E* **100**, 013108 (2019).

7. V. Iyer, H. Gaensbauer, T. L. Daniel, S. Gollakota, Wind dispersal of battery-free wireless

devices. *Nature* **603**, 427–433 (2022).

8. Y. Zhang, H. Guo, S. B. Kim, Y. Wu, D. Ostojich, S. H. Park, X. Wang, Z. Weng, R. Li, A. J. Bandodkar, Y. Sekine, J. Choi, S. Xu, S. Quaggin, R. Ghaffari, J. A. Rogers, Passive sweat collection and colorimetric analysis of biomarkers relevant to kidney disorders using a soft microfluidic system. *Lab. Chip* **19**, 1545–1555 (2019).

9. Y. Takahashi, S. Danwittayakul and T. M. Suzuki, Dithizone nanofiber-coated membrane for filtration-enrichment and colorimetric detection of trace Hg (II) ion *Analyst* **134**, 1380–1385 (2009).

10. W. Kurz, A. K. Yetisen, M. V. Kaito, M. J. Fuchter, M. Jakobi, M. Elsner, A. W. Koch, UV-sensitive wearable devices for colorimetric monitoring of UV exposure. *Adv. Opt. Mater.* **8**, 1901969 (2020).

11. M. Momtaz, J. Chen, High-performance colorimetric humidity sensors based on konjac glucomannan. *ACS Appl. Mater. Interfaces* **12**, 54104–54116 (2020).

12. K. Kwon, J. U. Kim, Y. Deng, S. R. Krishnan, J. Choi, H. Jang, K. H. Lee, C.-J. Su, I. Yoo, Y. Wu, L. Lipschultz, J.-H. Kim, T. S. Chung, D. Wu, Y. Park, T. Kim, R. Ghaffari, S. Lee, Y. Huang, J. A. Rogers, An on-skin platform for wireless monitoring of flow rate, cumulative loss and temperature of sweat in real time. *Nat. Electron.* **4**, 302–312 (2021).

13. Y. H. Jung, T.-H. Chang, H. Zhang, C. Yao, Q. Zheng, V. W. Yang, H. Mi, M. Kim, S. J. Cho, D.-W. Park, H. Jiang, J. Lee, Y. Qiu, W. Zhou, Z. Cai, S. Gong, Z. Ma, High-performance green flexible electronics based on biodegradable cellulose nanofibril paper. *Nat. Commun.* **6**, 7170 (2015).

14. J.-W. Shin, J. Chan Choe, J. H. Lee, W. B. Han, T.-M. Jang, G.-J. Ko, S. M. Yang, Y.-G. Kim, J. Joo, B. H. Lim, E. Park, S.-W. Hwang, Biologically safe, degradable self-destruction system for on-demand, programmable transient electronics. *ACS Nano* **15**, 19310–19320 (2021).

15. H. Fu, K. Nan, W. Bai, W. Huang, K. Bai, L. Lu, C. Zhou, Y. Liu, F. Liu, J. Wang, M. Han,

Z. Yan, H. Luan, Y. Zhang, Y. Zhang, J. Zhao, X. Cheng, M. Li, J. Woo Lee, Y. Liu, D. Fang, X. Li, Y. Huang, Y. Zhang, J. A. Rogers, Morphable 3D mesostructures and microelectronic devices by multistable buckling mechanics. *Nat. Mater.* **17**, 268–276 (2018).

16. Y. Park, H. Luan, K. Kwon, S. Zhao, D. Franklin, H. Wang, H. Zhao, W. Bai, J.-U. Kim, W. Lu, J.-H. Kim, Y. Huang, Y. Zhang, J. A. Rogers, Transformable, freestanding 3D mesostructures based on transient materials and mechanical interlocking. *Adv. Funct. Mater.* **29**, 1903181 (2019).

17. Z. Yan, F. Zhang, F. Liu, M. Han, D. Ou, Y. Liu, Q. Lin, X. Guo, H. Fu, Z. Xie, M. Gao, Y. Huang, J. H. Kim, Y. Qiu, K. Nan, J. Kim, P. Gutruf, H. Luo, A. Zhao, K.-C. Hwang, Y. Huang, Y. Zhang, J. A. Rogers, Mechanical assembly of complex, 3D mesostructures from releasable multilayers of advanced materials. *Sci. Adv.* **2**, e1601014 (2016).

18. M. Han, H. Wang, Y. Yang, C. Liang, W. Bai, Z. Yan, H. Li, Y. Xue, X. Wang, B. Akar, H. Zhao, H. Luan, J. Lim, I. Kandela, G. A. Ameer, Y. Zhang, Y. Huang, J. A. Rogers, Three-dimensional piezoelectric polymer microsystems for vibrational energy harvesting, robotic interfaces and biomedical implants. *Nat. Electron.* **2**, 26–35 (2019).

19. P. Pounds and S. Singh, Samara: Biologically inspired self-deploying sensor networks. *IEEE Potentials* **34**, 10–14 (2015).

20. J. M. Anderson, M. S. Shive, Biodegradation and biocompatibility of PLA and PLGA microspheres. *Adv. Drug Deliv. Rev.* **28**, 5–24 (1997).

21. X. Huang, Y. Liu, K. Chen, W.-J. Shin, C.-J. Lu, G.-W. Kong, D. Patnaik, S.-H. Lee, J. F. Cortes, J. A. Rogers, Stretchable, wireless sensors and functional substrates for epidermal characterization of sweat. *Small* **10**, 3083–3090 (2014).

22. N. Chigurupati, L. Saiki, C. Gayser, A. K. Dash, Evaluation of red cabbage dye as a potential natural color for pharmaceutical use. *Int. J. Pharm.* **241**, 293–299 (2002).

23. H. Torres-Pierna, D. Ruiz-Molina, C. Roscini, Highly transparent photochromic films with a

tunable and fast solution-like response. *Mater. Horiz.* **7**, 2749–2759 (2020).

24. M.-H. You, X. Yan, J. Zhang, X.-X. Wang, X.-X. He, M. Yu, X. Ning, Y.-Z. Long, Colorimetric humidity sensors based on electrospun polyamide/$CoCl_2$ nanofibrous membranes. *Nanoscale Res. Lett.* **12**, 360 (2017).

25. R. Smith, D. R. Sabatino, T. J. Praisner, Temperature sensing with thermochromic liquid crystals. *Exp. Fluids* **30**, 190–201 (2001).

26. P. I. P. Park, S. Jonnalagadda, Predictors of glass transition in the biodegradable poly-lactide and poly-lactide-co-glycolide polymers. *J. Appl. Polym. Sci.* **100**, 1983–1987 (2006).

27. B. H. Kim, J. Lee, S. M. Won, Z. Xie, J.-K. Chang, Y. Yu, Y. K. Cho, H. Jang, J. Y. Jeong, Y. Lee, A. Ryu, D. H. Kim, K. H. Lee, J. Y. Lee, F. Liu, X. Wang, Q. Huo, S. Min, D. Wu, B. Ji, A. Banks, J. Kim, N. Oh, H. M. Jin, S. Han, D. Kang, C. H. Lee, Y. M. Song, Y. Zhang, Y. Huang, K.-I. Jang, J. A. Rogers, Three-dimensional silicon electronic systems fabricated by compressive buckling process. *ACS Nano* **12**, 4164–4171 (2018).

28. Y. S. Choi, R. T. Yin, A. Pfenniger, J. Koo, R. Avila, K. Benjamin Lee, S. W. Chen, G. Lee, G. Li, Y. Qiao, A. Murillo-Berlioz, A. Kiss, S. Han, S. M. Lee, C. Li, Z. Xie, Y.-Y. Chen, A. Burrell, B. Geist, H. Jeong, J. Kim, H.-J. Yoon, A. Banks, S.-K. Kang, Z. J. Zhang, C. R. Haney, A. V. Sahakian, D. Johnson, T. Efimova, Y. Huang, G. D. Trachiotis, B. P. Knight, R. K. Arora, I. R. Efimov, J. A. Rogers, Fully implantable and bioresorbable cardiac pacemakers without leads or batteries. *Nat. Biotechnol.* **39**, 1228–1238 (2021).

29. Y. S. Choi, Y.-Y. Hsueh, J. Koo, Q. Yang, R. Avila, B. Hu, Z. Xie, G. Lee, Z. Ning, C. Liu, Y. Xu, Y. J. Lee, W. Zhao, J. Fang, Y. Deng, S. M. Lee, A. Vázquez-Guardado, I. Stepien, Y. Yan, J. W. Song, C. Haney, Y. S. Oh, W. Liu, H.-J. Yoon, A. Banks, M. R. MacEwan, G. A. Ameer, W. Z. Ray, Y. Huang, T. Xie, C. K. Franz, S. Li, J. A. Rogers, Stretchable, dynamic covalent polymers for soft, long-lived bioresorbable electronic stimulators designed to facilitate neuromuscular regeneration. *Nat. Commun.* **11**, 5990 (2020).

30. V.S. Bisaria, T.K. Ghose, Biodegradation of cellulosic materials: Substrates,

microorganisms, enzymes and products. *Enzyme Microbiol. Technol.* **3**, 90–104 (1981).

31. J.-T. Kim, Y. Jin, S. Shen, A. Dash, L. P. Chamorro, Free fall of homogeneous and heterogeneous cones. *Phys. Rev. Fluids* **5**, 093801 (2020).

32. C. Cummins, M. Seale, A. Macente, D. Certini, E. Mastropaolo, I. M. Viola, N. Nakayama, A separated vortex ring underlies the flight of the dandelion, *Nature* **562**, 414–418 (2018).

33. H. Li, X. Wang, F. Zhu, X. Ning, H. Wang, J. A. Rogers, Y. Zhang, Y. Huang, Viscoelastic characteristics of mechanically assembled three-dimensional structures formed by compressive buckling, *J. Appl. Mech.* **85**, 121002 (2018).

34. S. Vogel, *Life in Moving Fluids: The Physical Biology of Flow* (Princeton Univ. Press, 1981).

35. S. B. Kim, Y. Zhang, S. M. Won, A. J. Bandodkar, Y. Sekine, Y. Xue, J. Koo, S. W. Harshman, J. A. Martin, J. M. Park, T. R. Ray, K. E. Crawford, K.-T. Lee, J. Choi, R. L. Pitsch, C. C. Grigsby, A. J. Strang, Y.-Y. Chen, S. Xu, J. Kim, A. Koh, J. S. Ha, Y. Huang, S. W. Kim, J. A. Rogers, Super-absorbent polymer valves and colorimetric chemistries for time-sequenced discrete sampling and chloride analysis of sweat via skin-mounted soft microfluidics. *Small* **14**, e1703334 (2018).

36. A. Koh, D. Kang, Y. Xue, S. Lee, R. M. Pielak, J. Kim, T. Hwang, S. Min, A. Banks, P. Bastien, M. C. Manco, L. Wang, K. R. Ammann, K.-I. Jang, P. Won, S. Han, R. Ghaffari, U. Paik, M. J. Slepian, G. Balooch, Y. Huang, J. A. Rogers, A soft, wearable microfluidic device for the capture, storage, and colorimetric sensing of sweat. *Sci. Transl. Med.* **8**, 366ra165 (2016).

37. J. Kim, Y. Wu, H. Luan, D. S. Yang, D. Cho, S. S. Kwak, S. Liu, H. Ryu, R. Ghaffari, J. A. Rogers, A skin-interfaced, miniaturized microfluidic analysis and delivery system for colorimetric measurements of nutrients in sweat and supply of vitamins through the skin. *Adv. Sci.* **9**, 2103331 (2022).

38. E. Scanes, P. R. Scanes, P. M. Ross, Climate change rapidly warms and acidifies Australian

estuaries. *Nat. Commun.* **11**, 1803 (2020).

39. L. Li, L. F. Liao, Y. P. Ding, H. Y. Zeng, Dithizone-etched CdTe nanoparticles-based fluorescence sensor for the off–on detection of cadmium ion in aqueous media, *RSC Adv.* **7**, 10361–10368 (2017).

40. C. Tong, L. Tong, F. Jian, R. Brady, Photochromic wool fabrics from a hybrid silica coating. *Text. Res. J.* **77**, 923–928 (2007).

41. R.L. Stoddard, J.S. McIndoe, The color-changing sports drink: An ingestible demonstration *J. Chem. Educ.* **90**, 1032–1034 (2013).

42. Y. Takahashi, H. Kasai, H. Nakanishi, T. M. Suzuki, Test strips for heavy-metal ions fabricated from nanosized dye compounds, *Angew. Chem. Int. Ed.* **45**, 913–916 (2006).

43. R. F. Rachmadi, I. K. E. Rachmadi, Vehicle color recognition using convolutional neural network. arXiv:1510.07391 [cs.CV] (26 October 2015).

44. Mikołajczyk A., Grochowski M. Data augmentation for improving deep learning in image classification problem, in *Proceedings of the 2018 International Interdisciplinary PhD Workshop, IIPhDW* (IEEE, 2018), pp. 117–122.

45. L. Lu, C. A. Garcia, A. G. Mikos, In vitro degradation of thin poly(DL-lactic-co-glycolic acid) films, *J. Biomed. Mater. Res.* **46**, 236–244 (2017).

46. W. Thielicke, E. J. Stamhuis, T. William, R. Sonntag, Particle image velocimetry for MATLAB: Accuracy and enhanced algorithms in PIVlab, *J. Open Res. Softw.* **9**, 12 (2021).

47. American Society for Testing Materials (ASTM) Standard Test Method for Fungicides for Controlling Sapstain and Mold on Unseasoned Lumber (Laboratory Method), Standard D 4445-91. Annual Book of ASTM Standards, 4.10 (American Society for Testing Materials, 1998), pp. 497–500.

48. American Wood Protection Association Book of Standards, *Use Category System: User*

Specifications for Treated Wood (U1-14) (AWPA, 2014).

49. F. W. Roos, W. W. Willmarth, Some experimental results on sphere and disk drag. *AIAA J.* **9**, 285–291 (1971).

50. D. Lentink, W. B. Dickson, J. L. Van Leeuwen, M. H. Dickinson, Leading-edge vortices elevate lift of autorotating plant seeds. *Science* **324**, 1438–1440 (2009).

51. M. R. A. Nabawy, W. J. Crowther, On the quasi-steady aerodynamics of normal hovering flight part ii: Model implementation and evaluation. *J. R. Soc. Interface* **11**, 20131197 (2014).

52. M. R. A. Nabawy, W. J. Crowther, The role of the leading edge vortex in lift augmentation of steadily revolving wings: A change in perspective. *J. R. Soc. Interface* **14**, 20170159 (2017).

53. H. Schlichting, E. Truckenbrodt, *Aerodynamics of the Airplane* (McGraw-Hill Companies, 1979).

54. J. Roskam, C. T. E. Lan, *Airplane Aerodynamics and Performance* (DAR Corporation, 1997).

55. G. R. Spedding, J. McArthur, Span efficiencies of wings at low Reynolds numbers. *J. Aircr.* **47**, 120–128 (2010).

56. H. Liu, "Aerodynamic characteristics of flat plate airfoil at low Reynolds numbers," thesis, Johns Hopkins University (2021).
